# Supplementary material for: Adaptive design of mRNA-loaded extracellular vesicles for targeted immunotherapy of cancer
Source: Nat Commun. 2023 Oct 19;14:6610. doi: 10.1038/s41467-023-42365-5 (PMC10587228; doi:10.1038/s41467-023-42365-5)
Supplement: Supplementary file 1 — Supplementary Information [file 41467_2023_42365_MOESM1_ESM.pdf]

TABLE OF CONTENTS

1. Supplemental figures and legends

Fig. S1 | Schematic representation of the nanosecond pulse electroporation (nsEP) system

Fig. S2 | Plasmid loading and mRNA transcription analysis

Fig. S3 | High-throughput generation of small extracellular vesicles (sEVs) by the nsEP system

Fig. S4 | Optimization of nsEP conditions for MEFs

Fig. S5 | Morphological characterization of sEVs using microscopy

Fig. S6 | Western blot analysis of sEVs generated from different preparations

Fig. S7 | Optimization of nsEP conditions for HEK293T cells

Fig. S8 | Evaluation of stably transfected cell line

Fig. S9 | Western blot analysis of sEV pull-down assay

Fig. S10 | Characterization of target mRNA encapsulation in sEVs

Fig. S11 | Evaluation of CD71 expression in GBM cell lines

Fig. S12 | Evaluation of the binding capacity of CD64-sEV

Fig. S13 | Evaluation of imsEV binding capacity using flow cytometry

Fig. S14 | Characterization of EVs produced by HEK293T cells

Fig. S15 | Biocompatibility evaluation

Fig. S16 | Cellular uptake of imsEV in GL261 cell

Fig. S17 | Analysis of imsEV uptake pathway in GL261 cells

Fig. S18 | Detection of INF-γ concentration in cell culture supernatant

Fig. S19 | Hemolysis test

Fig. S20 | Biosafety evaluation

Fig. S21 | Tissue distribution analysis of imsEV in healthy mice

Fig. S22 | Blood cell counts after a single injection of the indicated preparations

Fig. S23 | Characterization of T cells in the blood of naïve mice after repeated injections of the indicated preparations

Fig. S24 | Characterization of T cells in the spleens of naïve mice after repeated injections of the indicated preparations

Fig. S25 | Infiltration of immune cells in the GL261 model

Fig. S26 | Evaluation of the therapeutic effect of imsEVs in the GL261 model

Fig. S27 | Biosafety evaluation of imsEVs in the GL261 model

Fig. S28 | Characterization of IFN-γ in residual GBM tumour tissues

Fig. S29 | Characterization of MHC-I in residual GBM tumour tissues

Fig. S30 | Characterization of $CD8^+$ cells in tumour tissues

Fig. S31 | Characterization of $CD86^+$ macrophages in tumour tissues

Fig. S32 | Characterization of $Iba1^+$ activated mononuclear in tumours

Fig. S33 | Total blood cell counts in the SB28 tumour-bearing mouse model

Fig. S34 | Characterization of T cells in the blood of SB28 tumour-bearing mice

Fig. S35 | Characterization of T cells in the spleens of SB28 tumour-bearing mice

Fig. S36 | Evaluation of the therapeutic effect of imsEV in the SB28 model

Fig. S37 | Biosafety evaluation of imsEV in SB28 model

Fig. S38 | Flow chart for estimating copy numbers of plasmids and subsequently transcribed mRNA in transfected cells

Fig. S39 | Representative flow cytometry gating strategies

2. Supplemental Table

Table S1 | Controlling sEV release from MEFs by adjusting the amplitude of nanosecond pulses

Table S2 | Controlling sEV release from MEFs by adjusting the duration of nanosecond pulses

Table S3 | Controlling sEV release from MEFs by adjusting the frequency of nanosecond pulses

Table S4 | Controlling sEV release from HEK293T cells by adjusting the amplitude of nanosecond pulses

Table S5 | Controlling sEV release from HEK293T cells by adjusting the duration of nanosecond pulses

Table S6 | Controlling sEV release from HEK293T cells by adjusting the frequency of nanosecond pulses

Table S7 | Changes in sEV-related proteins in mouse embryonic fibroblasts after nsEP treatment

Table S8 | Differentially expressed proteins associated with sEV secretion based on protein-protein interaction analysis

Table S9 | Top 95 proteins differentially expressed after nsEP treatment

Table S10 | sEV incubated with the indicated antibody ratios of monoclonal anti-CD71 and anti-PDL1 antibodies, followed by measurement of binding of imsEV to anti-CD71 and anti-PDL1 antibodies by tethered lipoplex nanoparticle (TLN) technology

SUPPLEMENTAL FIGURES AND LEGENDS

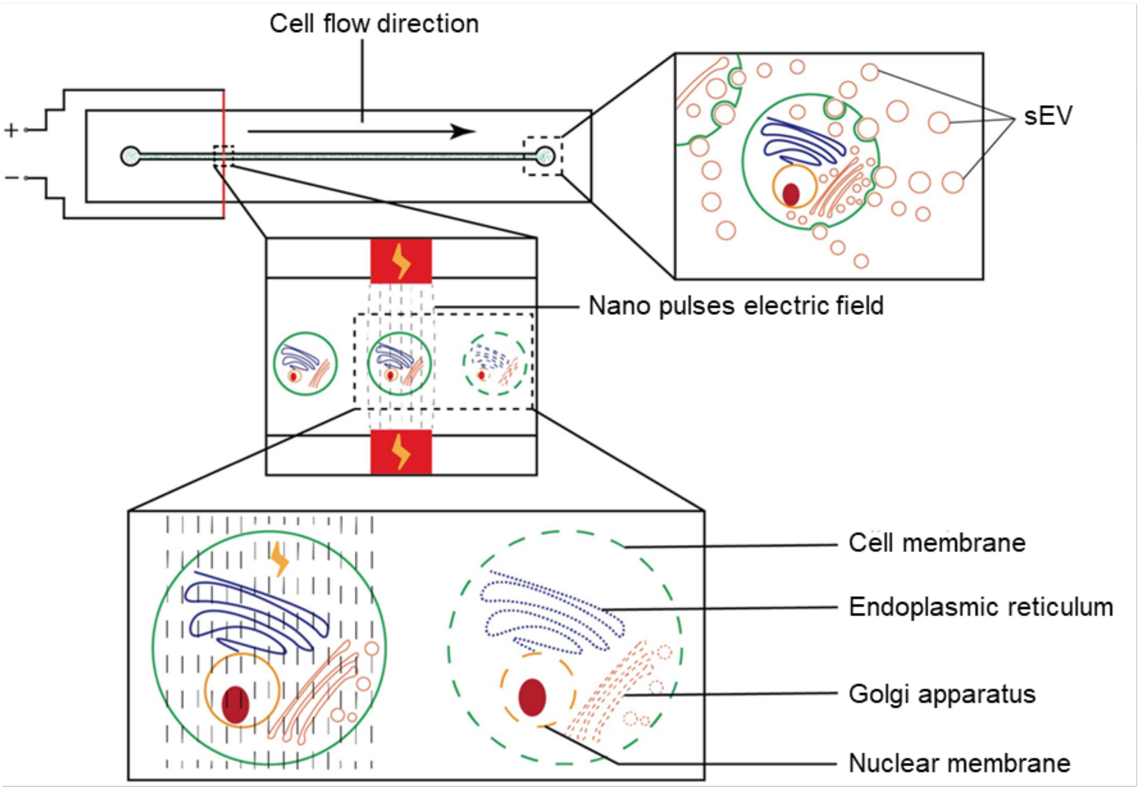

Supplementary Fig. 1 | Schematic representation of the nanosecond pulse electroporation (nsEP) system. The nsEP system facilitates plasmid transfection to cross both the plasma membrane and nuclear membrane.

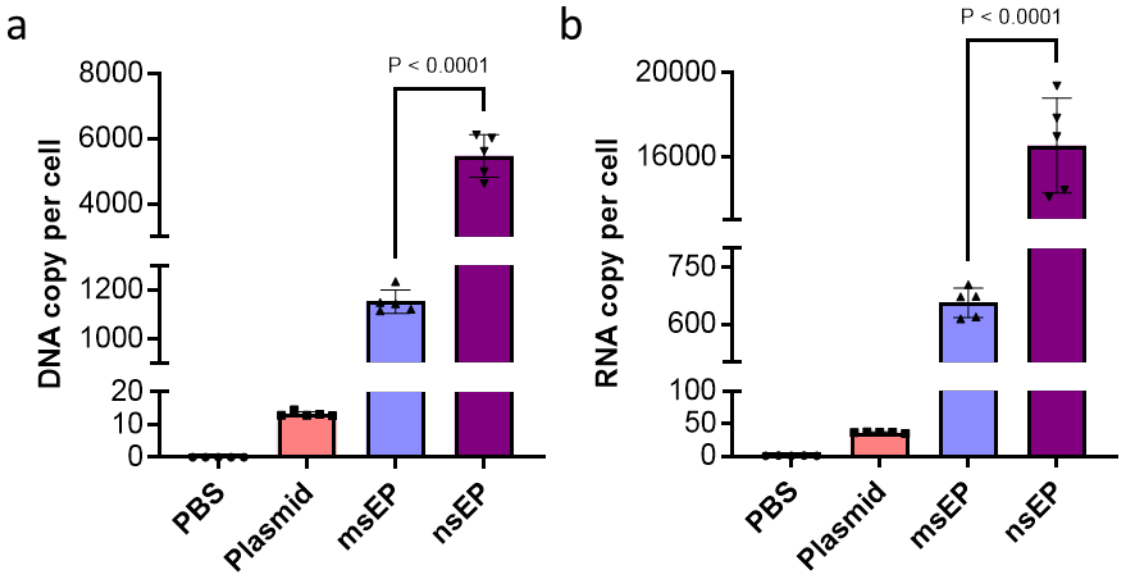

Supplementary Fig. 2 | Plasmid loading and mRNA transcription analysis. a, The copy number of DNA or, **b,** RNA in cells is determined by correlating the Ct (cycle threshold) values of RT-qPCR. The copy number in each sample is then divided by the cell number (calculated based on the cell viability data) from which DNA was extracted to receive the copies of plasmid (or RNA) per cell. In detail, around 5600 copies of target plasmids were averagely loaded in each cell 3 hours after the transfection, and around 16000 copies of the target mRNA were transcribed after another three hours. In contrast, millisecond electroporation pulse (msEP) supplied only around 1200 copies of DNA and 650 copies of RNA, respectively. Data are presented as means ± standard deviation (SD) for n = 5 biologically independent samples; statistical significance was calculated by one-way analysis of variance with Tukey's multiple comparisons test. Source data are provided as a Source data file.

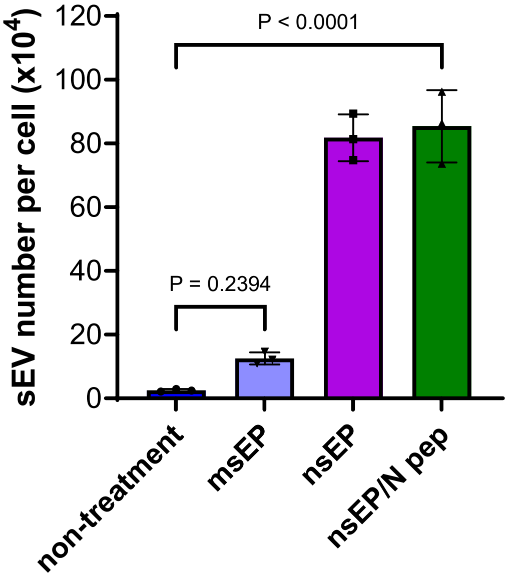

Supplementary Fig. 3 | High-throughput generation of small extracellular vesicles (sEVs) by the nsEP system. Numbers of sEVs per cell produced by human embryonic kidney 293T (HEK293T) cells in untreated control (non-treatment), msEP, nsEP, and nsEP plus N peptide treatment groups. Data are presented as means ± SD for n = 3 biologically independent samples; statistical significance was calculated by one-way analysis of variance with Tukey's multiple comparisons test. Source data are provided as a Source data file.

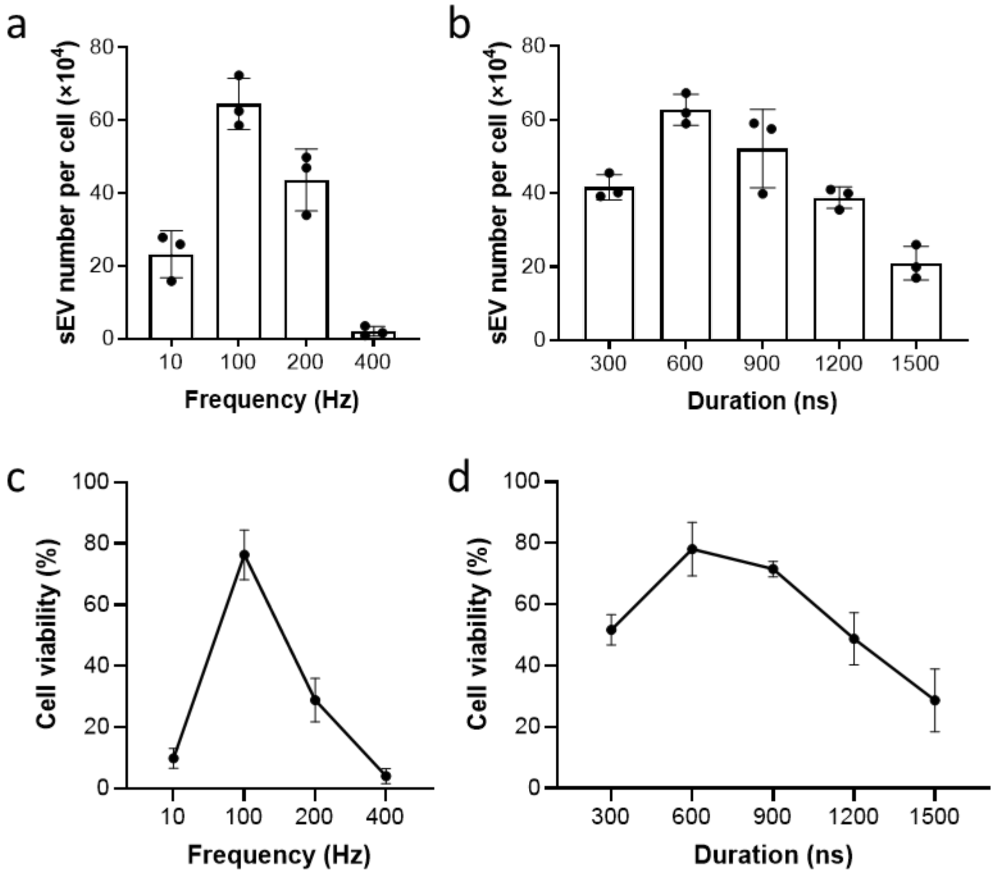

Supplementary Fig. 4 | Optimization of nsEP conditions for MEFs. a, Numbers of sEVs per cell produced by mouse embryonic fibroblasts (MEFs) after nsEP followed by msEP at frequencies from 10 to 400 Hz. **b,** The quantity of sEV per cell produced by MEFs by nsEP with pulses lasting from 300 to 1500 ns. **c,** Viability of MEFs after nsEP at voltage amplitudes from 10 to 400 Hz. **d,** Viability of MEFs after nsEP at pulse durations of 300 to 1500 ns. Data in **a, b, c,** and **d** are presented as means ± SD, n = 3 biologically independent samples. Source data are provided as a Source data file.

.

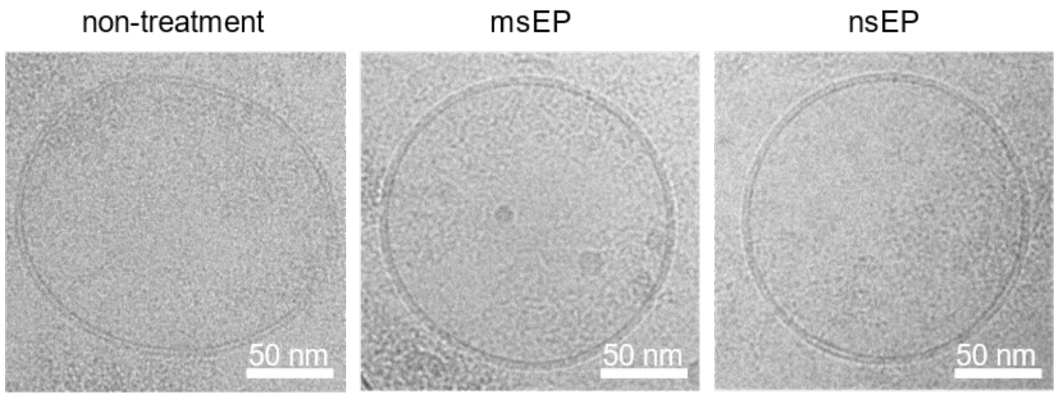

Supplementary Fig. 5 | Morphological characterization of sEVs using microscopy. Cyro-electron microscopic images of non-treatment (control) sEV and sEV generated by msEP or nsEP show typical vesicle shape and size. The TEM images were representative data from three independent experiments.

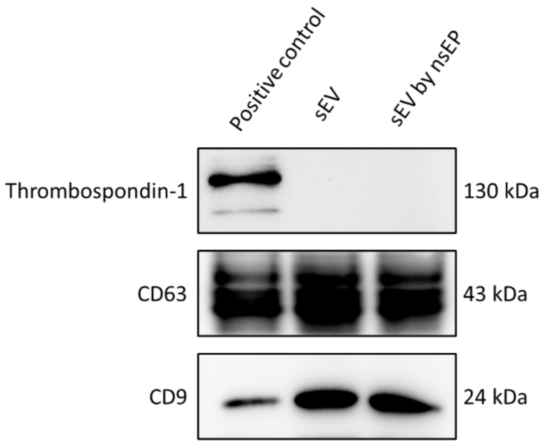

Supplementary Fig. 6 | Western blot analysis of sEVs generated from different preparations. Western blot assessment of thrombospondin-1, CD63, and CD9 expression in positive control, native sEVs, and sEVs based on nsEP electrical stimulation. As a positive control for apoptotic bodies, the supernatant of MEFs cultured in PBS for 48 hours was collected, followed by ultracentrifugation (100,000 × *g* for 2 h). The immunoblots were representative data from three independent experiments. Source data are provided as a Source data file.

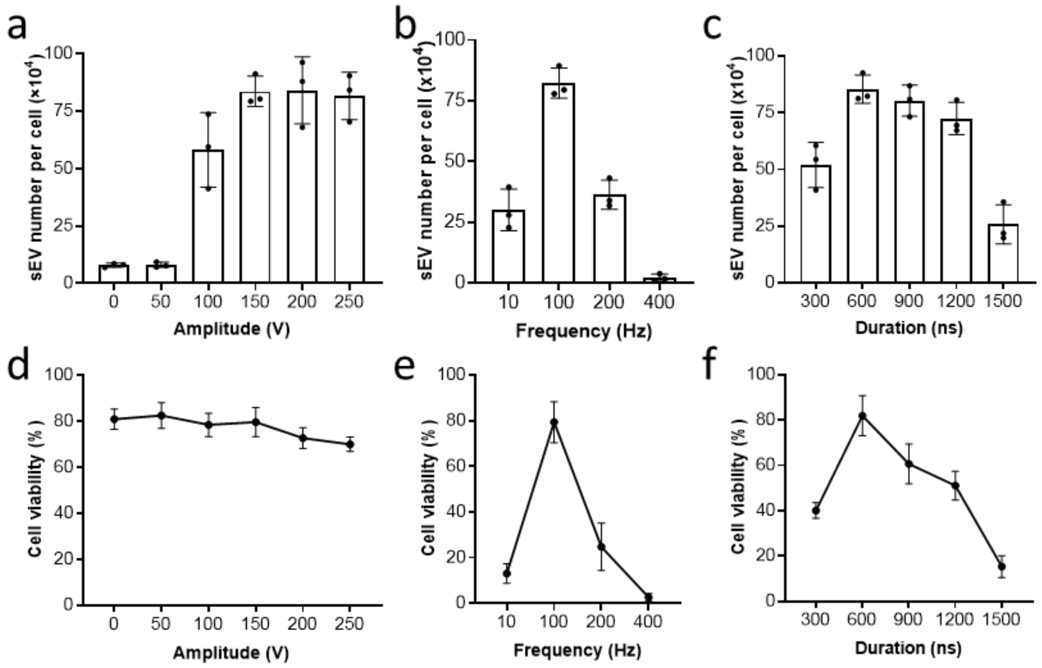

Supplementary Fig. 7 | Optimization of nsEP conditions for HEK293T cells. a, sEV numbers per cell produced by HEK293T cells by nsEP system at amplitudes from 0 to 250 V. **b,** The quantity of sEV per cell produced by HEK293T cells after nsEP system at frequencies from 10 to 400 Hz. **c,** sEV numbers per cell produced by HEK293T cells by nsEP system at durations from 300 to 1500 ns. **d,** Viability of HEK293T cells after nsEP at voltage amplitudes from 0 to 250 V. **e,** Viability of HEK293T cells after nsEP at frequencies from 10 to 400 Hz. **f,** Viability of HEK293T cells after nsEP at durations from 300 to 1500 ns. Data in **a, b**, **c**, **d**, **e,** and **f** are presented as means ± SD, n = 3 biologically independent samples. Source data are provided as a Source data file.

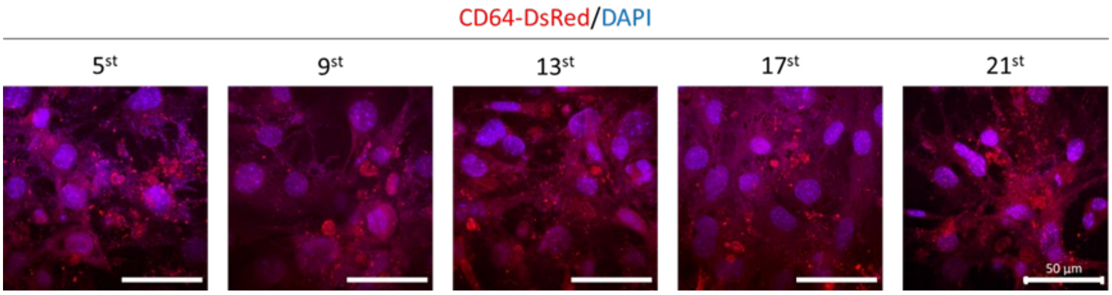

Supplementary Fig. 8 | Evaluation of stably transfected cell line. Confocal images depict the expression of CD64-DsRed in MEFs across different generations. Scale bar: 50 µm. The images were representative data from three independent experiments.

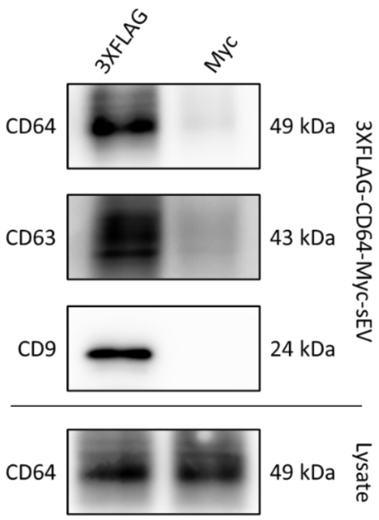

Supplementary Fig. 9 | Western blot analysis of sEV pull-down assay. The Western blots of an sEV pull-down assay show that FLAG beads successfully pull down the N-terminal FLAG of 3XFLAG-CD64-Myc, while Myc beads fail to pull down the C-terminal FLAG of 3XFLAG-CD64-Myc, suggesting that the N terminus of CD64 is located on the outside of sEVs while the C terminus of CD64 is positioned on the inside of sEVs. The immunoblots were representative data from three independent experiments. Source data are provided as a Source data file.

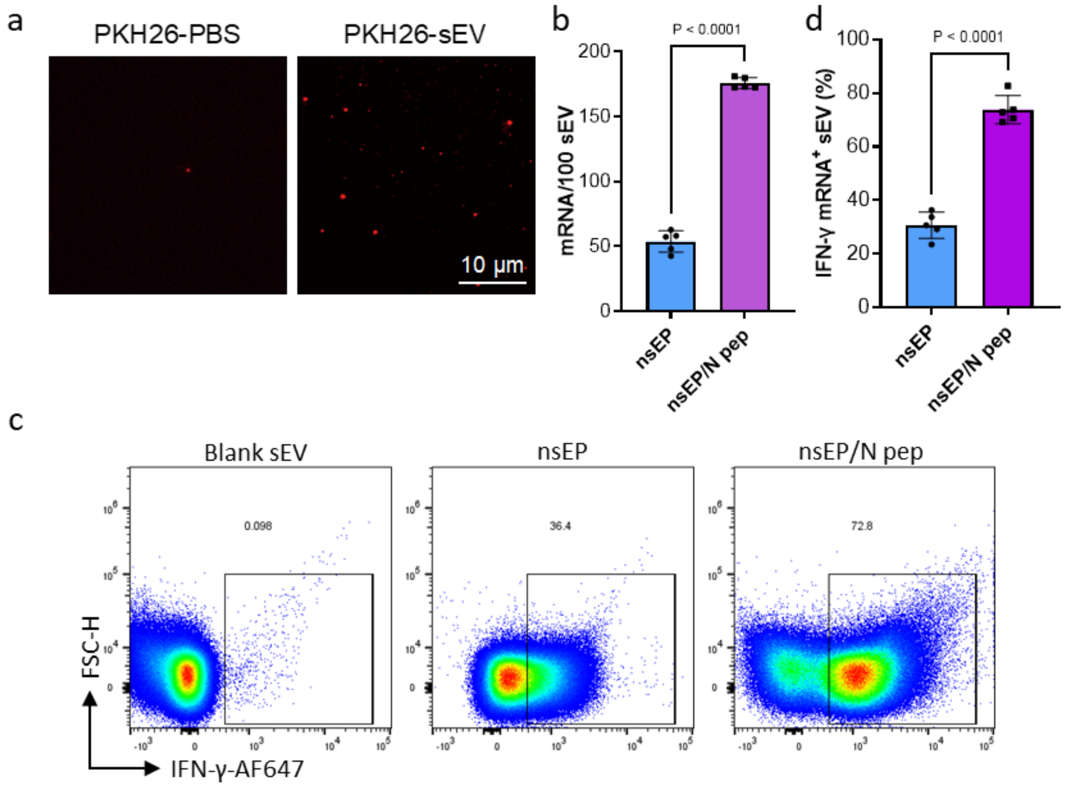

Supplementary Fig. 10 | Characterization of target mRNA encapsulation in sEVs. a, Representative total internal reflection fluorescence (TIRF) images of PKH26-PBS and PKH26-sEVs after the purification process. Scale bar: 10 μm. **b,** Quantification of the copy number of target mRNA in each EV sample using qPCR. The copy number/100 sEV is obtained by dividing the mRNA copy number by the sEV number. **c,** Representative fluorescence data obtained for plain sEV, nsEP, and nsEP/N pep (left to right) stained with PKH26 and IFN-γ molecular beacon. Focusing on PKH26-positive vesicles, the nsEP/N pep demonstrated the highest colocalization rate (72.8% compared to 36.4% for the nsEP and 0.098% for blank sEVs). **d,** Quantification of the percentage of IFN-γ positive fluorescent events expressed on PKH26-positive EVs in nsEP and nsEP/N pep preparations. The colocalization efficiency was higher in the nsEP/N pep condition compared to the nsEP condition. Data in **b** and **d** represent means ± SD, n = 5 biologically independent samples; statistical significance was analyzed with unpaired two-tailed Student's *t*-tests. The TIRF images and flow cytometry were representative data from five independent experiments. Source data are provided as a Source data file.

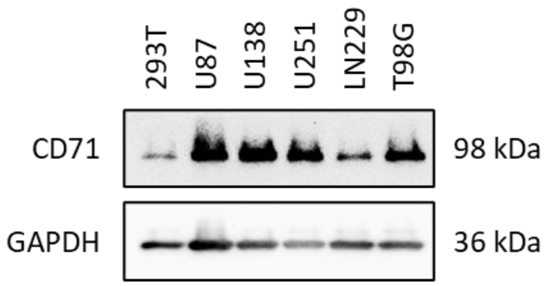

Supplementary Fig. 11 | Evaluation of CD71 expression in GBM cell lines. Western blot reveals CD71 expression in five human GBM cell lines. The HEK293T cell line was included as a negative control. The immunoblots were representative data from three independent experiments. Source data are provided as a Source data file.

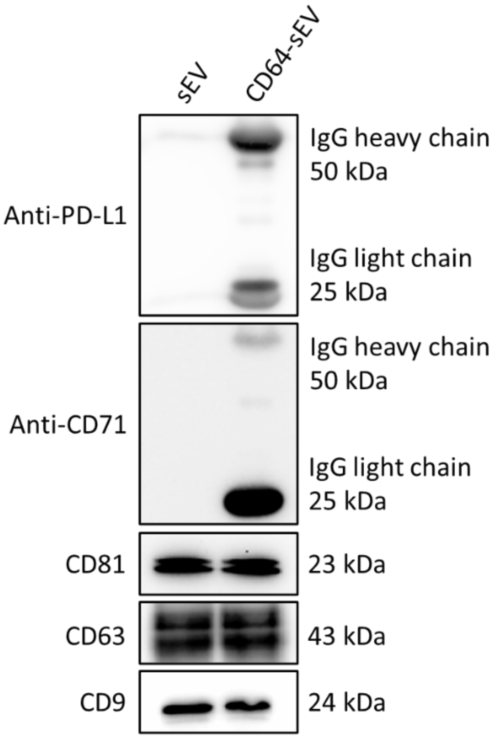

Supplementary Fig. 12 | Evaluation of the binding capacity of CD64-sEV. Western blots demonstrate the ability of CD64-sEV to simultaneously bind Anti-PD-L1 and Anti-CD71. The immunoblots were representative data from three independent experiments. Source data are provided as a Source data file.

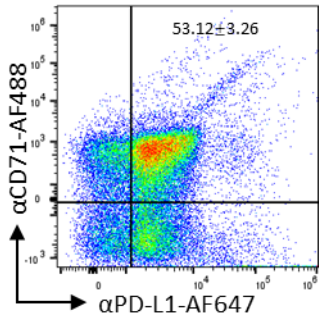

Supplementary Fig. 13 |Evaluation of imsEV binding capacity using flow cytometry. Representative fluorescence data obtained for imsEVs stained with PKH26, anti-PD-L1, and anti-CD71. Focusing on double-positive vesicles (anti-CD71 and anti-PD-L1), we showed that ~53.12 ± 3.26% of the double-positive events were detected above the fluorescence background in the PKH26-positive imsEV samples. This observation serves as evidence for the ability of imsEVs to effectively bind with functional anti-CD71 and anti-PD-L1. Although the efficiency of colocalization varied, we hypothesized that these differences primarily stemmed from the static and dynamic movement of imsEVs during their detection with different platforms. Data represent means ± SD, n = 5 biologically independent samples. The flow cytometry plot was representative data from three independent experiments.

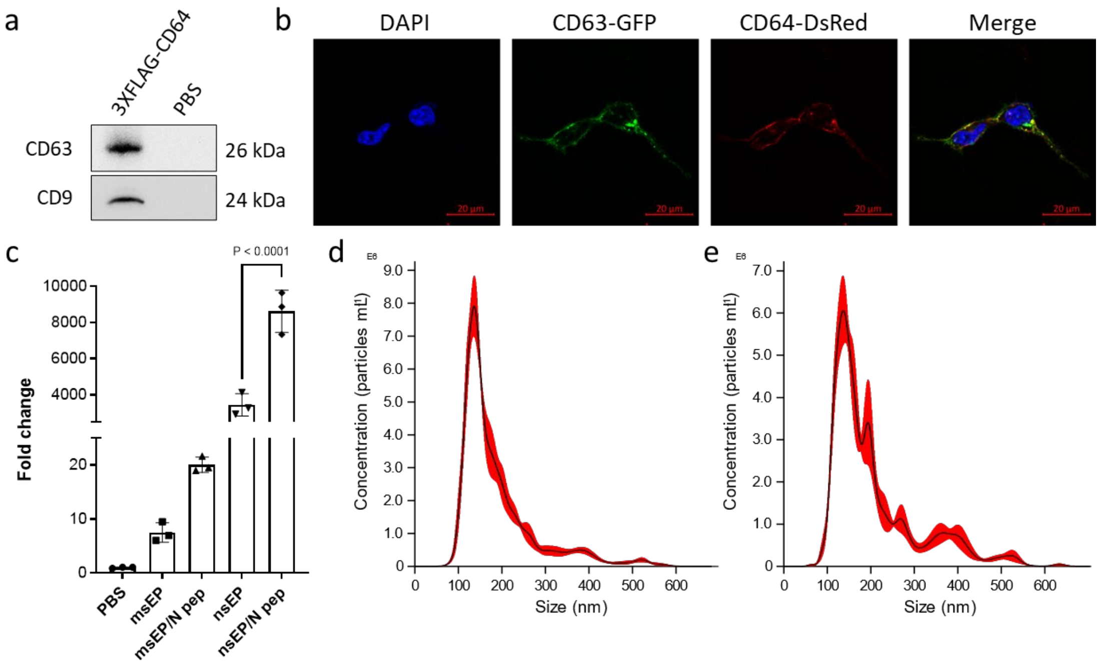

Supplementary Fig. 14 | Characterization of EVs produced by HEK293T cells. a, Western blots of an sEV pull-down assay show that FLAG beads could "pull down" the N-terminal FLAG of 3XFLAG-CD64, suggesting that the N terminus of CD64 is on the outside of the sEVs released by HEK293T cells. **b,** Confocal images of HEK293T cells transfected simultaneously with CD64-DsRed and CD63-GFP show extensive colocalization. Scale bar: 20 µm. **c,** CD64-N pep was co-transfected with box B-INF-γ or control INF-γ construct into HEK293T cells; imsEV were pelleted via ultracentrifugation. RT-qPCR was done on the imsEV and on the transfected cells for INF-γ and for a control mRNA (U6). **d, e,** Nanosight measurements of vesicle size distribution before (**d**) and after (**e**) incubation of CD64-sEV with IgG. A slight increase in imsEV size was observed relative to CD64-sEV released by HEK293T cells. Data in **c** are presented as means ± SD, n = 3 biologically independent samples; statistical significance was calculated by one-way analysis of variance with Tukey's multiple comparisons test. The immunoblots and confocal images were representative data from three independent experiments. Source data are provided as a Source data file.

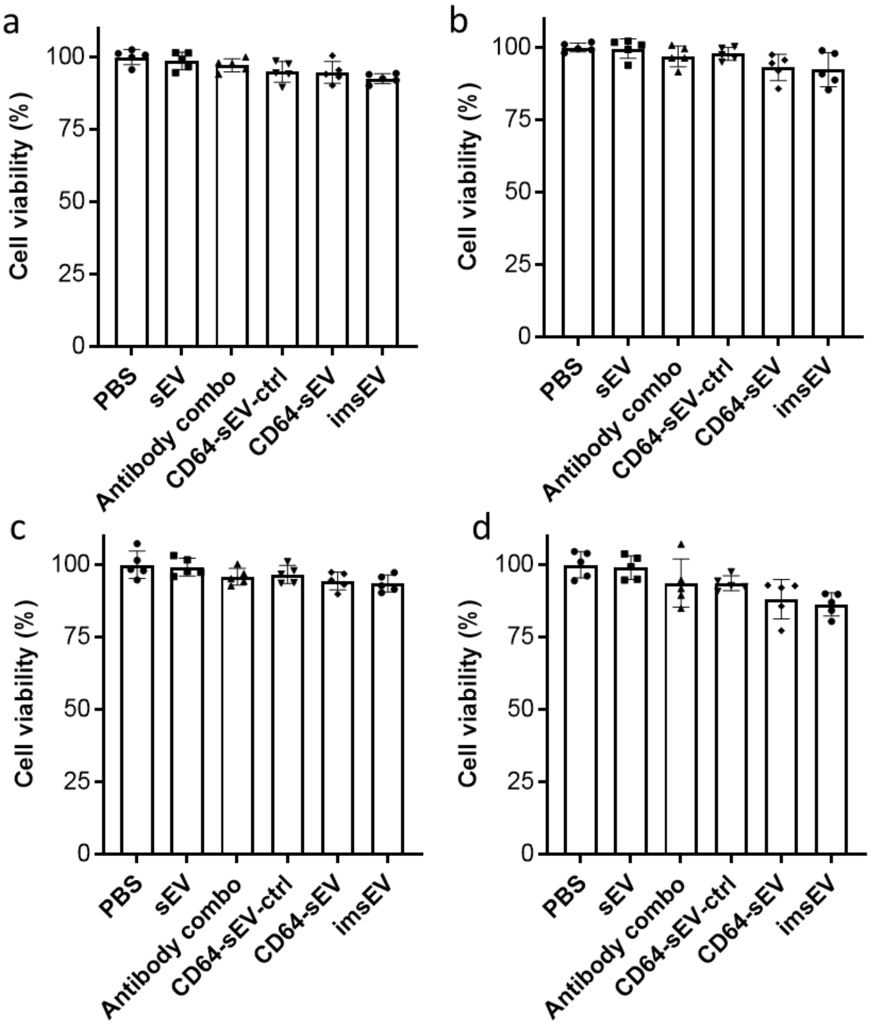

Supplementary Fig. 15 | Biocompatibility evaluation. a, b, Viability of SB28 cells treated with PBS, sEV, Antibody combo, CD64-sEV-ctrl, CD64-sEV, and imsEV after 24 h or 48 h. **c, d,** Viability of GL261 cells at 24 or 48 h after treatment with PBS, sEV, Antibody combo, CD64-sEV-ctrl, CD64-sEV, and imsEV. These findings suggest that imsEV has good biocompatibility. Data in **a, b**, **c**, and **d** are presented as means ± SD, n = 5 biologically independent samples. Source data are provided as a Source data file.

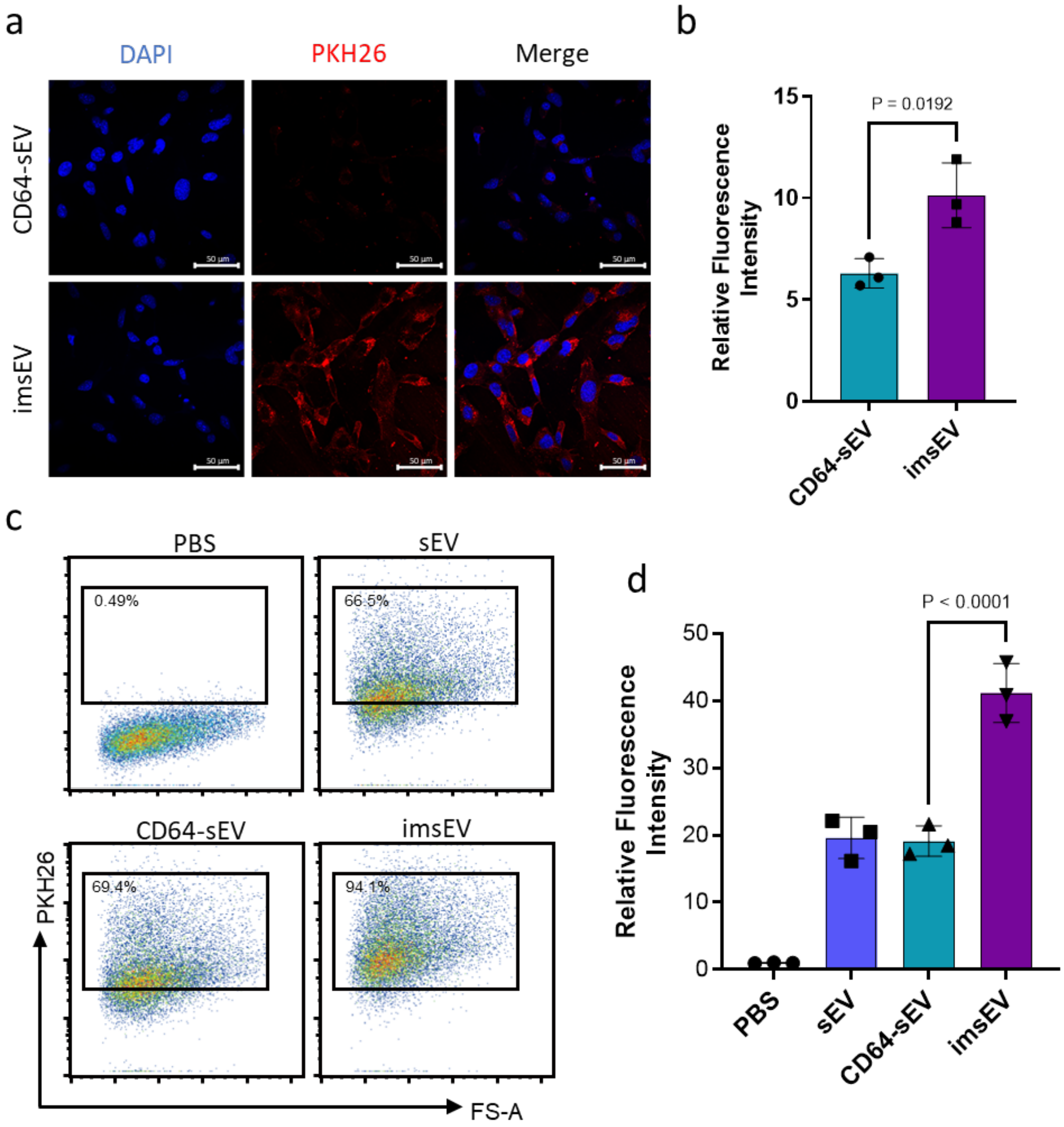

Supplementary Fig. 16 | Cellular uptake of imsEV in GL261 cells. a, b, Increased uptake of nsEP system-generated sEVs coated with CD71 antibody linked to CD64 by glioma (GL261) cells. Scale bar: 50 μm. **c, d,** Fluorescence intensity of PKH26-labeled sEVs taken up by GL261 cells measured by flow cytometry, confirming that imsEV uptake was the highest in GL261 cells. Data in **b** and **d** are presented as means ± SD, n = 3 biologically independent samples; statistical significance was calculated by one-way analysis of variance with Tukey's multiple comparisons test. The images and flow cytometry were representative data from three independent experiments. Source data are provided as a Source data file.

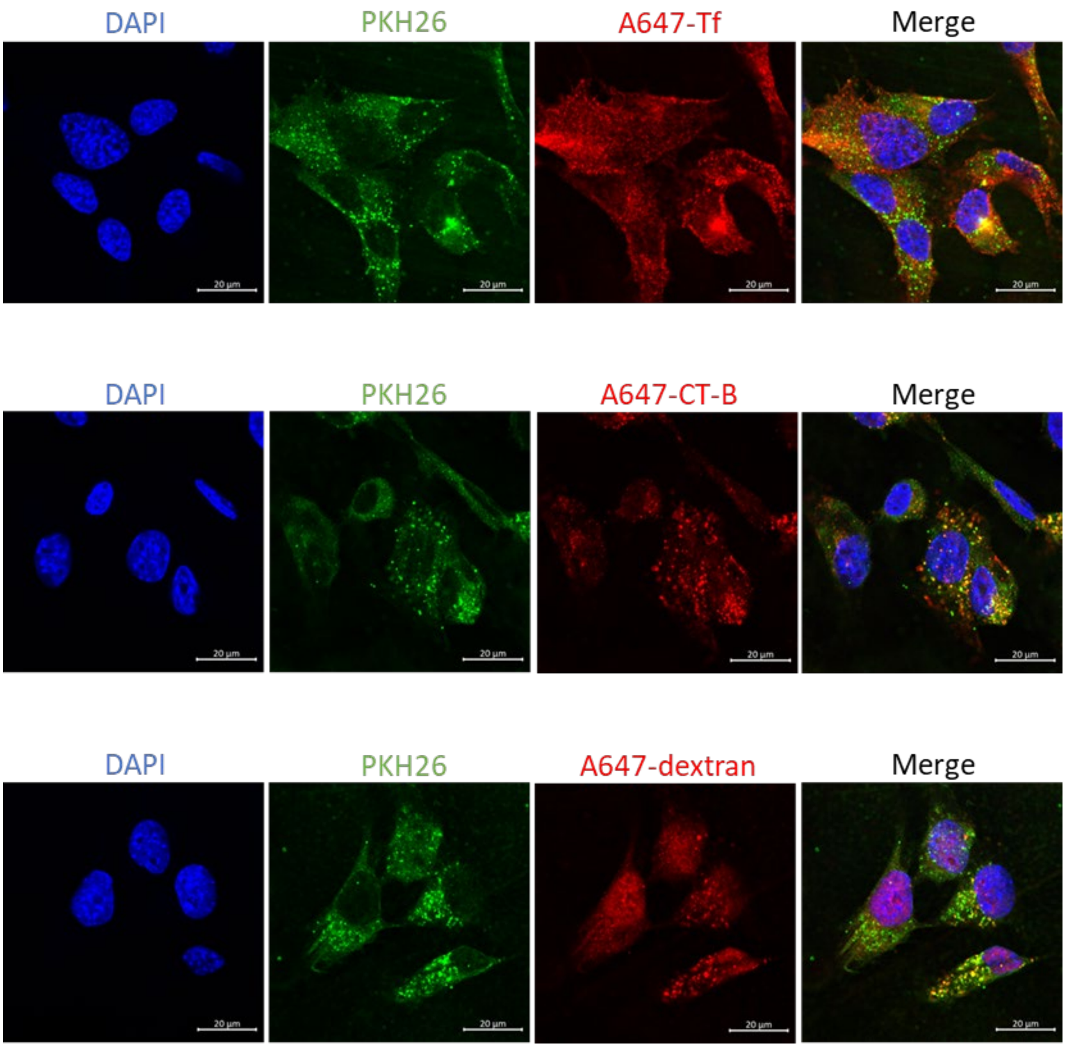

Supplementary Fig. 17 | Analysis of imsEV uptake pathway in GL261 cells.

Representative immunostainings of GL261 cells show colocalization of PKH26-labeled imsEV (green) with the indicated endocytosis markers (red). Most of the imsEVs were colocalized with transferrin-Alexa Fluor 647 (A647-Tf), suggesting that imsEVs are mainly taken up through clathrin-dependent endocytosis. A647-Tf is a marker of clathrin-dependent endocytosis; cholera toxin subunit B-Alexa Fluor 647 (A647-CT-B) is a marker of caveolae-dependent endocytosis; and A647-dextran is a marker of macropinocytosis. Scale bar: 20 μm. The images were representative data from three independent experiments.

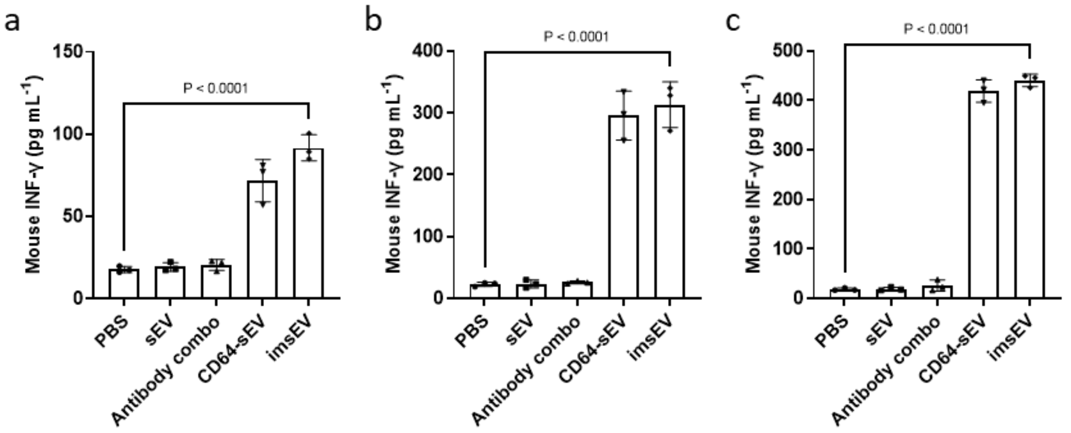

Supplementary Fig. 18 | Detection of INF-γ concentration in cell culture supernatant. a, INF-γ levels in the supernatant of SB28 cell culture medium after incubation with the indicated sEV preparations for 24 h, detected by ELISA. **b, c,** INF-γ levels in the supernatant of GL261 cell culture medium after incubation with the indicated sEV preparations for 24 h or 48 h, detected by ELISA. Data in **a, b**, and **c** are presented as means ± SD, n = 3 biologically independent samples; statistical significance was calculated by one-way analysis of variance with Tukey's multiple comparisons test. Source data are provided as a Source data file.

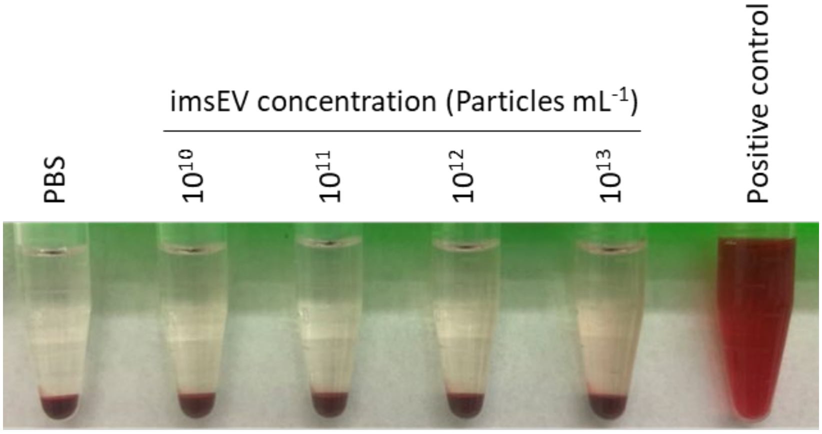

Supplementary Fig. 19 | Hemolysis test. Photograph of hemolysis of red blood cells treated *ex vivo* with imsEV. The red blood cells treated with 5% Triton X-100 were included as a positive control. The images were representative data from three independent experiments.

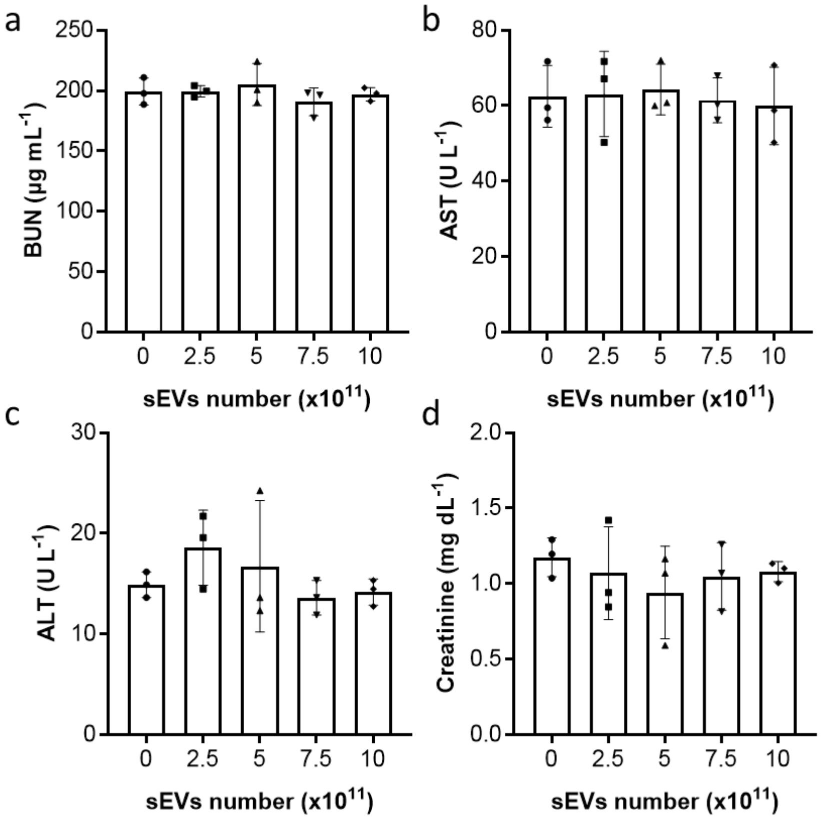

Supplementary Fig. 20 | Biosafety evaluation. a, b, c, d, Levels of blood urea nitrogen (BUN), aspartate transaminase (AST), alanine transaminase (ALT), and creatinine (measured by ELISA) in mice given different doses of imsEV. All data are presented as mean ± SD, n = 3 biologically independent samples. Source data are provided as a Source data file.

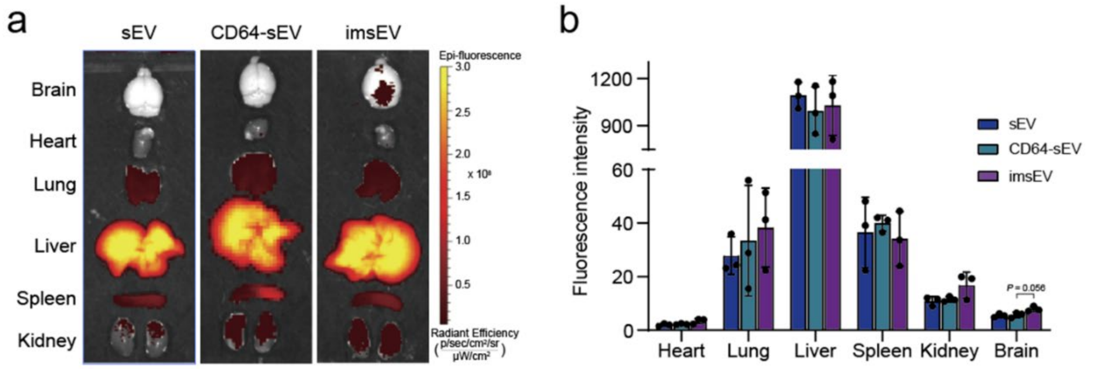

Supplementary Fig. 21 | Tissue distribution analysis of imsEV in healthy mice. a, b, Tissue distribution analyses in health mice indicated that imsEV showed increased brain targeting (n = 3, biologically independent samples), analyzed by one-way analysis of variance with Tukey's multiple comparisons test. Source data are provided as a Source data file.

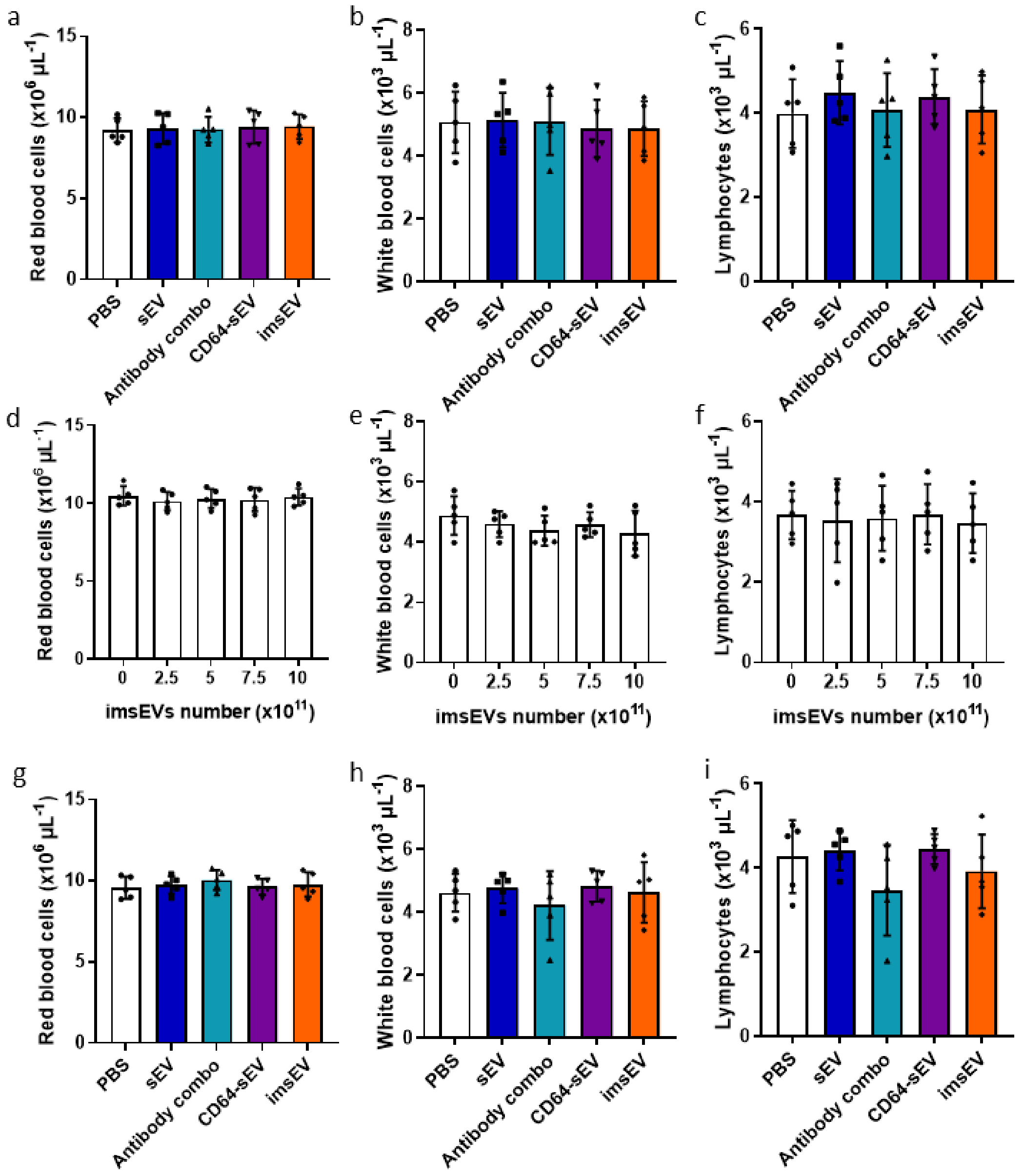

Supplementary Fig. 22 | Blood cell counts after a single injection of the indicated preparations. a, Red blood cell, **b,** White blood cell, and **c,** Lymphocyte counts after a single injection of the indicated preparations, **d,** Red blood cell, **e,** White blood cell, and **f,** Lymphocyte counts in mice after a single injection of different doses of imsEVs. **g,** Red blood cell, **h,** White blood cell, and **i,** Lymphocyte counts after a single injection of the indicated preparations. All data are presented as mean ± SD, n = 5 biologically independent samples, analyzed by one-way analysis of variance with Tukey's multiple comparisons test. Source data are provided as a Source data file.

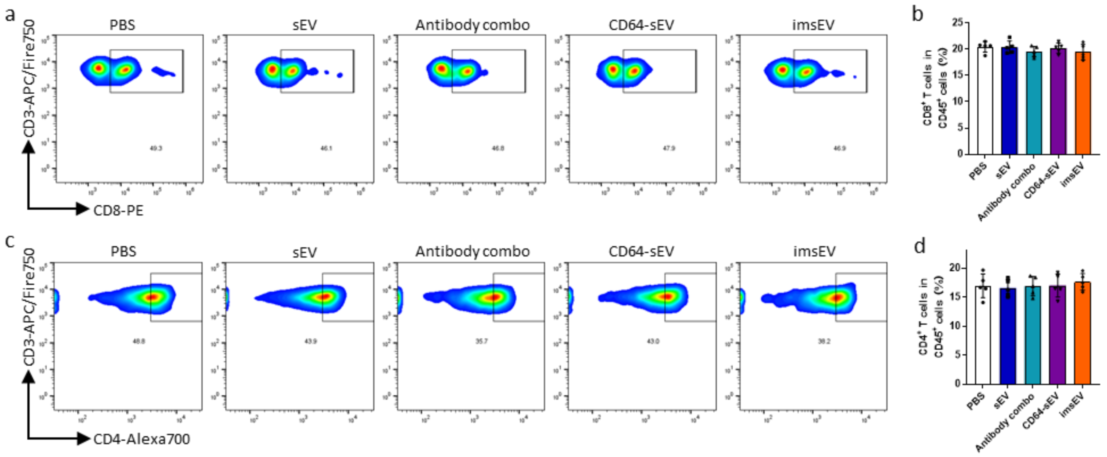

Supplementary Fig. 23 | Characterization of T cells in the blood of naïve mice after repeated injections of the indicated preparations. a, Representative plots of $CD8^+$ T cells (gated on $CD3^+$ cells) in blood after 5 injections of the indicated substances and analyzed by flow cytometry. **b,** Quantitative analysis of $CD8^+$ T cells in blood. **c,** Representative plots of $CD4^+$ T cells (gated on $CD3^+$ cells) in blood after 5 injections of the indicated substances and analyzed by flow cytometry. **d,** Quantitative analysis of $CD4^+$ T cells in blood. All data are presented as mean ± SD, n = 5 biologically independent samples, analyzed by one-way analysis of variance with Tukey's multiple comparisons test. Source data are provided as a Source data file.

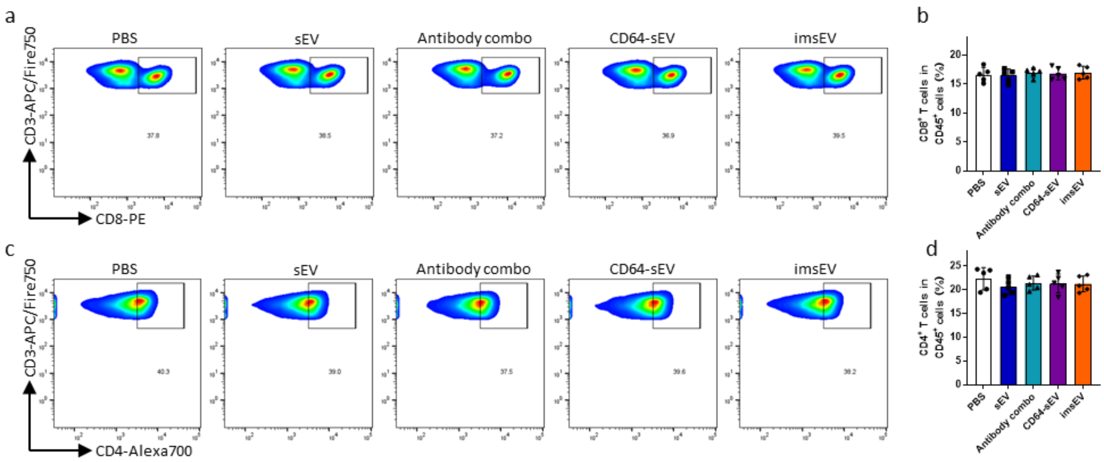

Supplementary Fig. 24 | Characterization of T cells in the spleens of naïve mice after repeated injections of the indicated preparations. a, Representative plots of CD8^{+} T cells (gated on CD3^{+} cells) in spleen after 5 injections of the indicated substances and analyzed by flow cytometry. **b,** Quantitative analysis of CD8^{+} T cells in spleen. **c,** Representative plots of CD4^{+} T cells (gated on CD3^{+} cells) in spleen after 5 injections of the indicated substances and analyzed by flow cytometry. **d,** Quantitative analysis of CD4^{+} T cells in spleen. All data are presented as mean ± SD, n = 5 biologically independent samples, analyzed by one-way analysis of variance with Tukey's multiple comparisons test. Source data are provided as a Source data file.

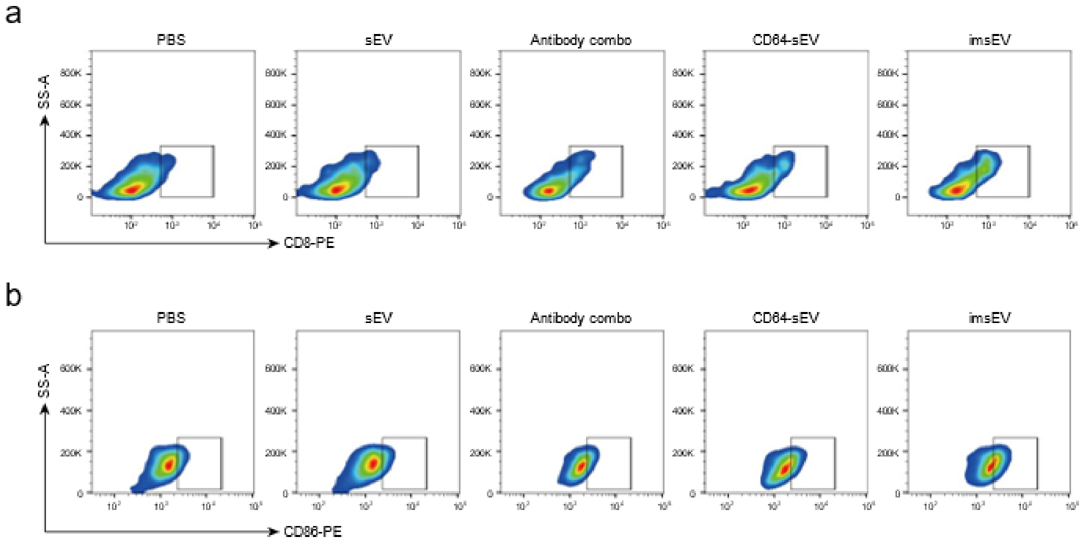

Supplementary Fig. 25 | Infiltration of immune cells in the GL261 model. a, Representative plots of T cells (gated on CD3$^+$ cells) in tumours after treatment with the indicated substances and analyzed by flow cytometry. **b,** Representative plots of macrophages (gated on F4/80$^+$ cells) in tumours after treatment with the indicated substances and analyzed by flow cytometry.

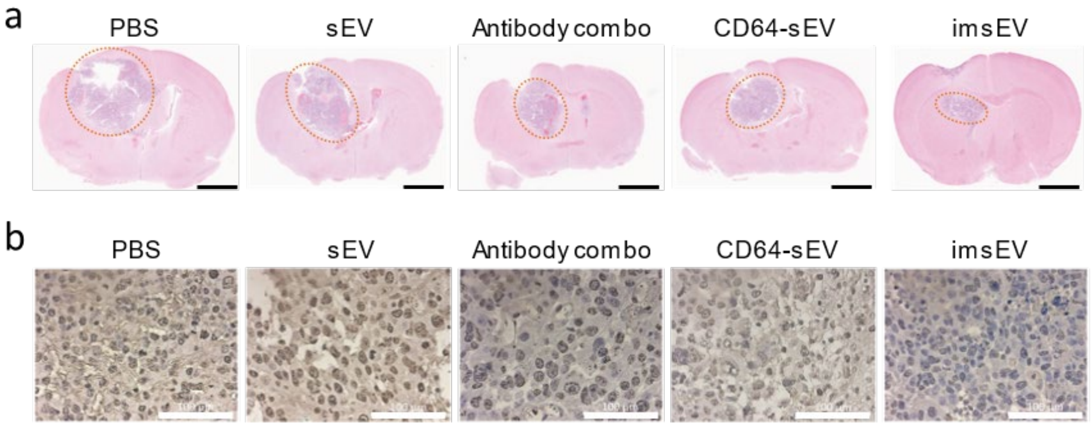

Supplementary Fig. 26 | Evaluation of the therapeutic effect of imsEVs in the GL261 model. a, H&E (Scale bar: 2 mm) and **b,** Ki67 staining of residual GL261 tumour tissue (Scale bar: 100 μm) from the indicated treatment groups show that imsEVs inhibited tumour cell proliferation in tumour tissues.

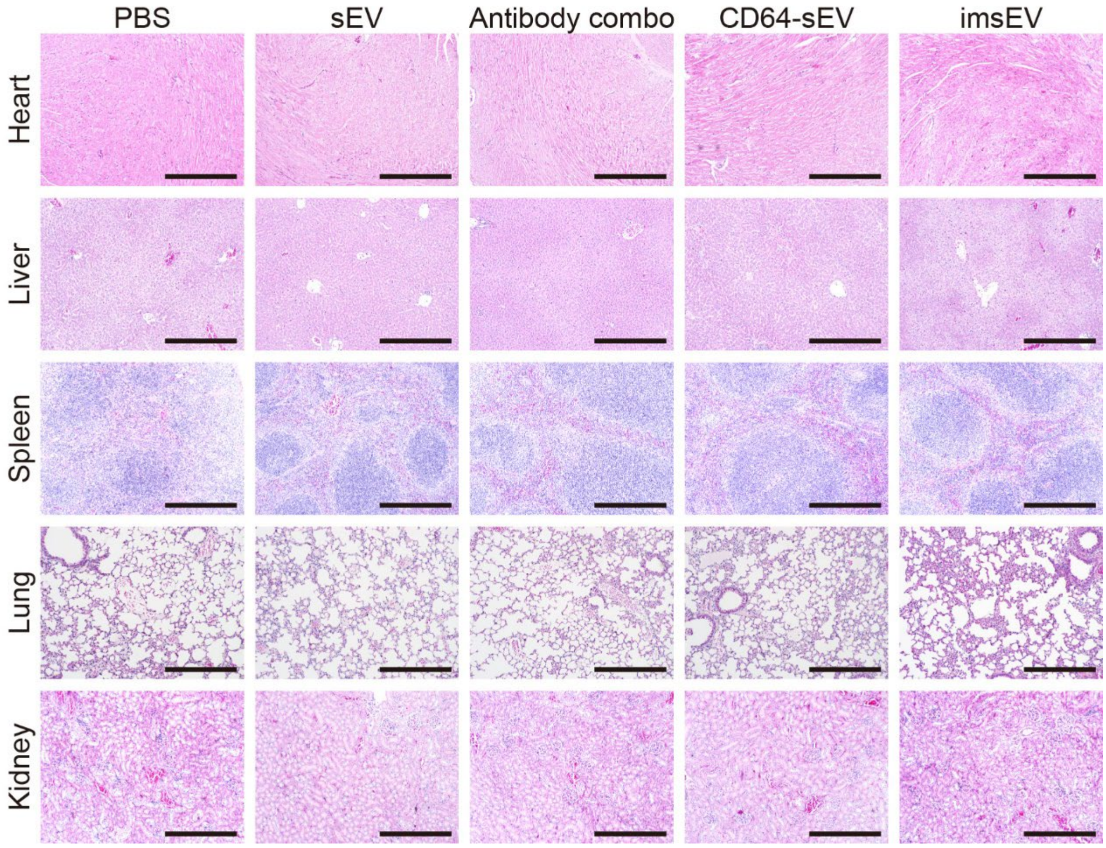

Supplementary Fig. 27 | Biosafety evaluation of imsEVs in the GL261 model. H&E staining of heart, liver, spleen, lung, and kidney tissues from the indicated treatment groups in the GL261 mouse model show that imsEVs had no detectable effect on the tissues examined. Scale bar: 500 μm.

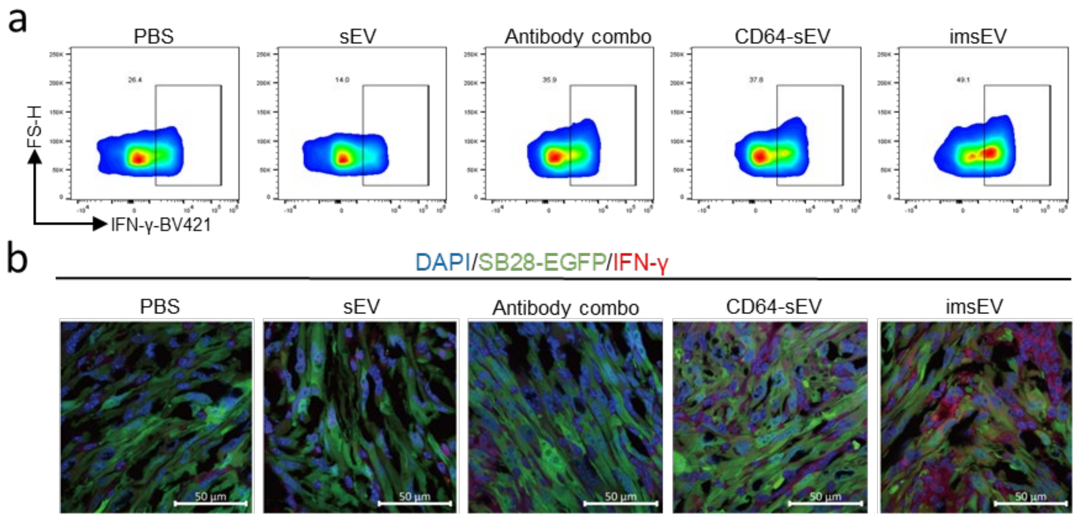

Supplementary Fig. 28 | Characterization of IFN-γ in residual GBM tumour tissues. a, Flow cytometry, **b,** Confocal assessment of IFN-γ staining in residual GBM tumour tissue from the indicated treatment groups showed that imsEV increased the expression of IFN-γ. Scale bar: 50 μm.

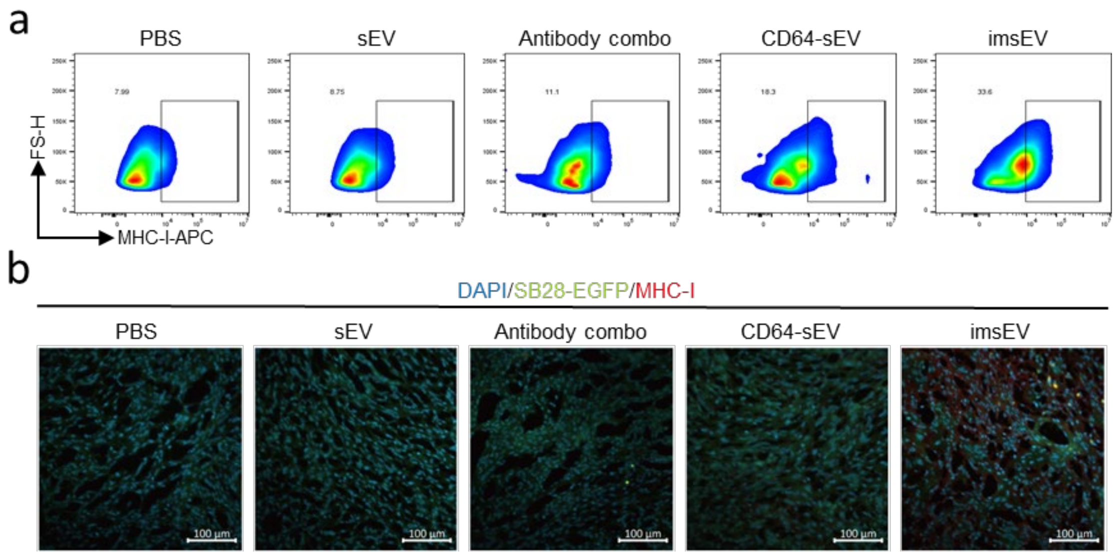

Supplementary Fig. 29 | Characterization of MHC-I in residual GBM tumour tissues. a, Flow cytometry, **b,** Confocal assessment of MHC-I staining in residual GBM tumour tissue from the indicated treatment groups showed that imsEV increased the expression of MHC-I. Scale bar: 100 μm.

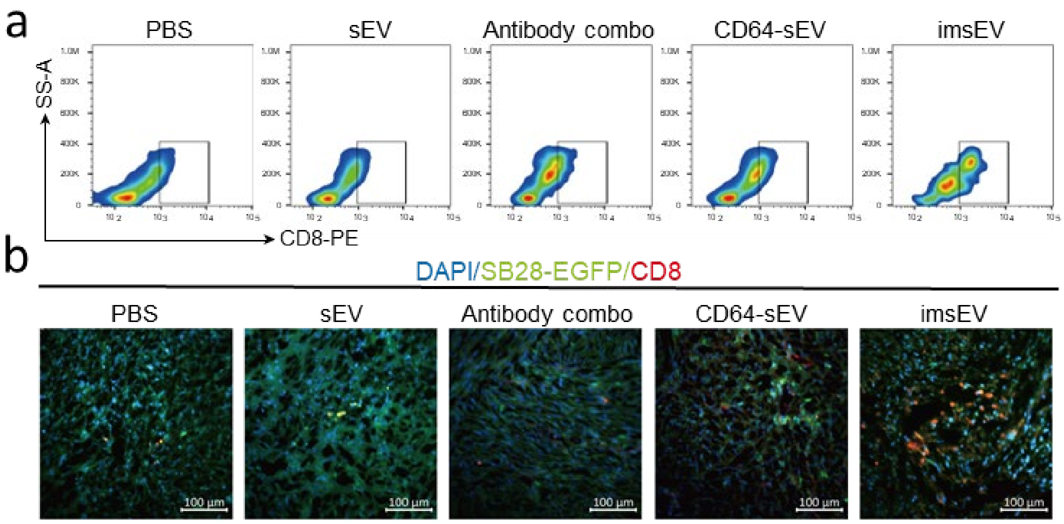

Supplementary Fig. 30 | Characterization of CD8$^+$ cells in tumour tissues. a, Flow cytometry, **b,** Immunofluorescence assessment of proportions of CD8$^+$ cells in tumour tissues of mice in the indicated treatment groups showed that imsEV led to increased proportions of CD8$^+$ cells. Scale bar: 100 μm.

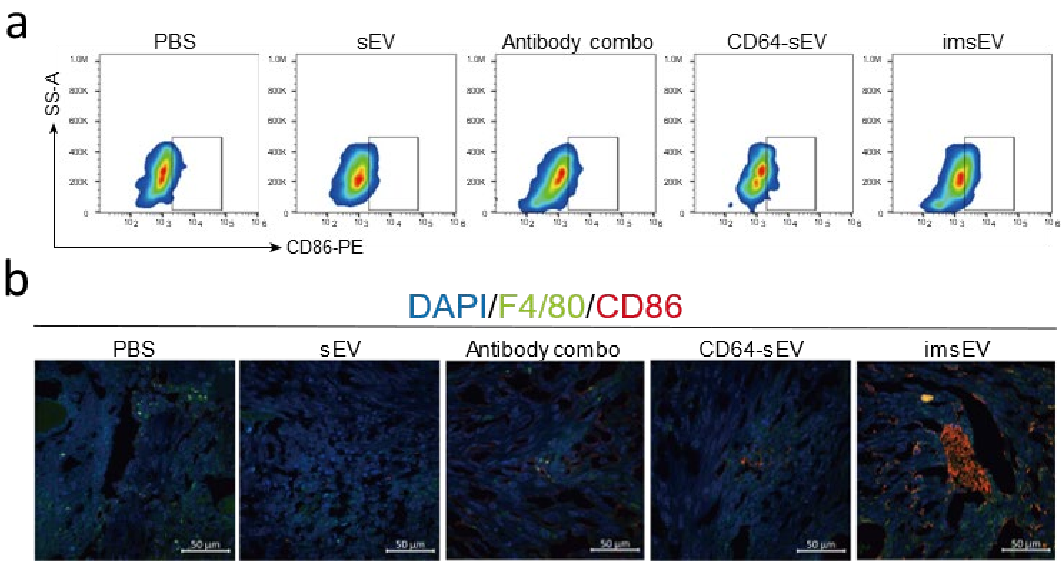

Supplementary Fig. 31 | Characterization of CD86$^+$ macrophages in tumour tissues. a, Flow cytometry, **b,** Immunofluorescence assessment of proportions of CD86$^+$ macrophages in tumour tissues of mice in the indicated treatment groups showed that imsEV led to increased proportions of CD86$^+$ macrophages. Scale bar: 50 μm.

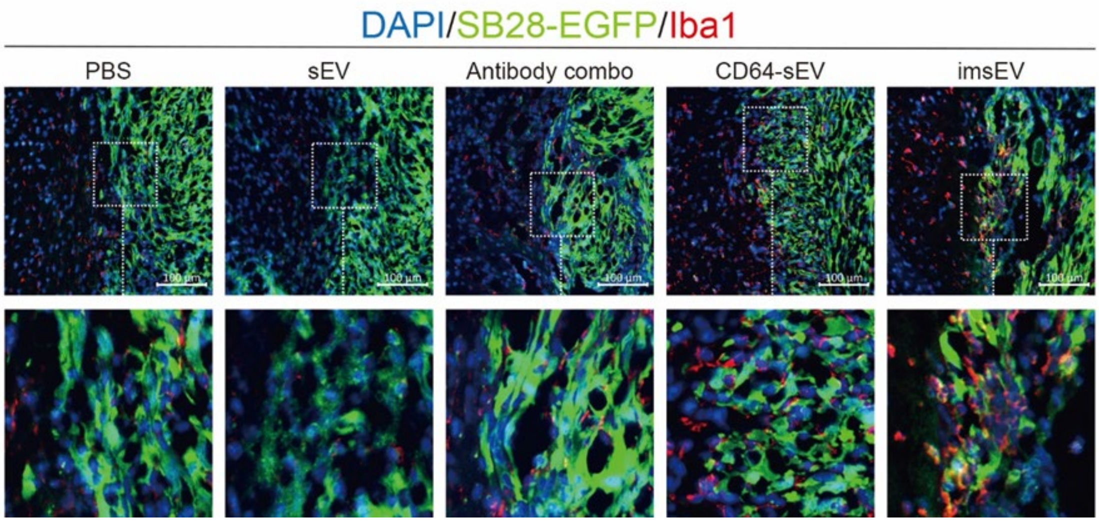

Supplementary Fig. 32 | Characterization of Iba1$^+$ activated mononuclear in tumours. The imsEV treatment significantly increased the number of Iba1$^+$ activated mononuclear cells within tumours. Scale bar: 100 μm.

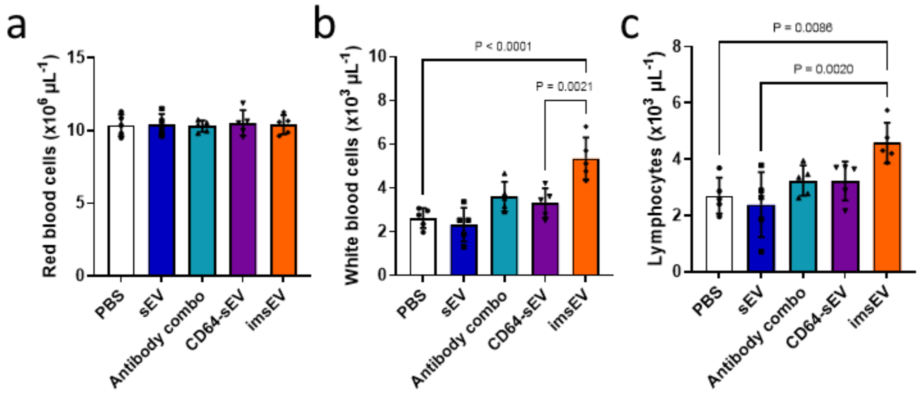

Supplementary Fig. 33 | Total blood cell counts in the SB28 tumour-bearing mouse model. a, Red blood cell, **b,** White blood cell, and **c,** Lymphocyte counts after five injections of the indicated preparations in SB28 tumour-bearing mice. (n = 5 biologically independent samples, analyzed by one-way analysis of variance with Tukey's multiple comparisons test). Source data are provided as a Source data file.

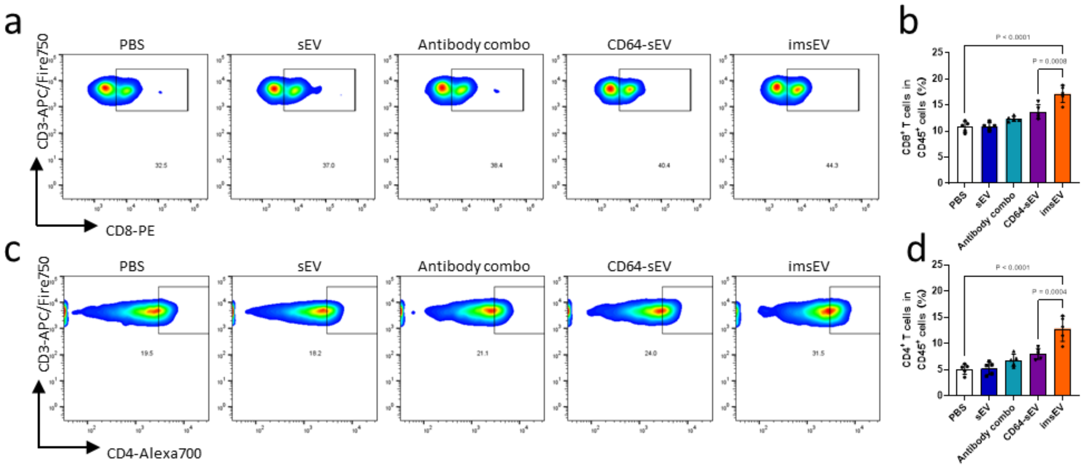

Supplementary Fig. 34 | Characterization of T cells in the blood of SB28 tumour-bearing mice. a, Representative plots of CD8^{+} T cells (gated on CD3^{+} cells) in blood after 5 injections with the indicated substances and analyzed by flow cytometry. **b,** Quantitative analysis of CD8^{+} T cells in blood. **c,** Representative plots of CD4^{+} T cells (gated on CD3^{+} cells) in blood after 5 injections with the indicated substances and analyzed by flow cytometry. **d,** Quantitative analysis of CD4^{+} T cells in blood. (n = 5 biologically independent samples, analyzed by one-way analysis of variance with Tukey's multiple comparisons test). Source data are provided as a Source data file

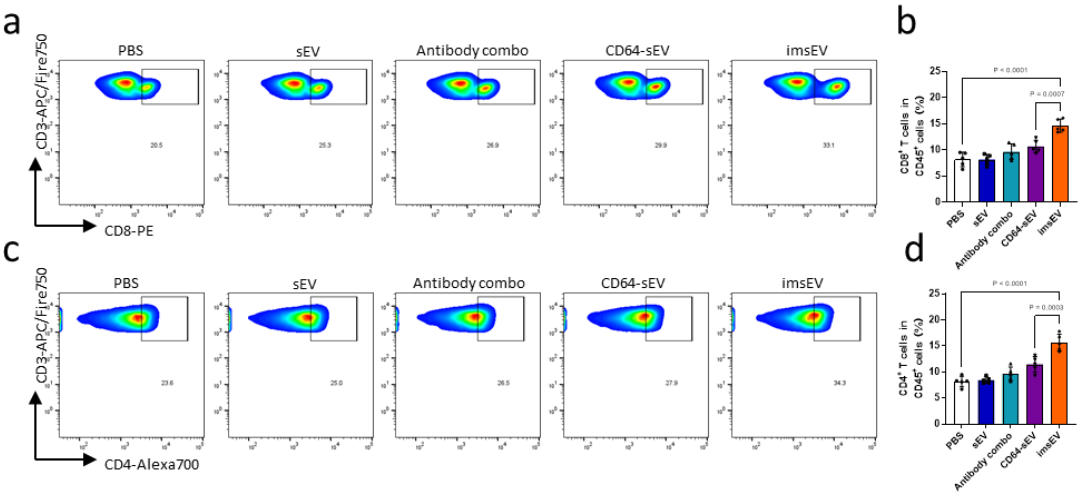

Supplementary Fig. 35 | Characterization of T cells in the spleens of SB28 tumour-bearing mice. a, Representative plots of $CD8^+$ T cells (gated on $CD3^+$ cells) in spleen after 5 injections with the indicated substances and analyzed by flow cytometry. **b,** Quantitative analysis of $CD8^+$ T cells in spleen. **c,** Representative plots of $CD4^+$ T cells (gated on $CD3^+$ cells) in spleen after 5 injections with the indicated substances and analyzed by flow cytometry. **d,** Quantitative analysis of $CD4^+$ T cells in spleen. (n = 5 biologically independent samples, analyzed by one-way analysis of variance with Tukey's multiple comparisons test). Source data are provided as a Source data file.

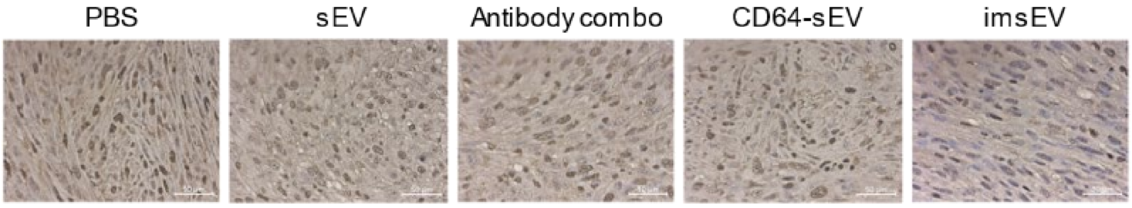

Supplementary Fig. 36 | Evaluation of the therapeutic effect of imsEV in the SB28 model. Ki67 stains of residual SB28 tumour tissues after the indicated treatments show that imsEV inhibited cell proliferation in tumour tissue. Scale bar: 50 µm.

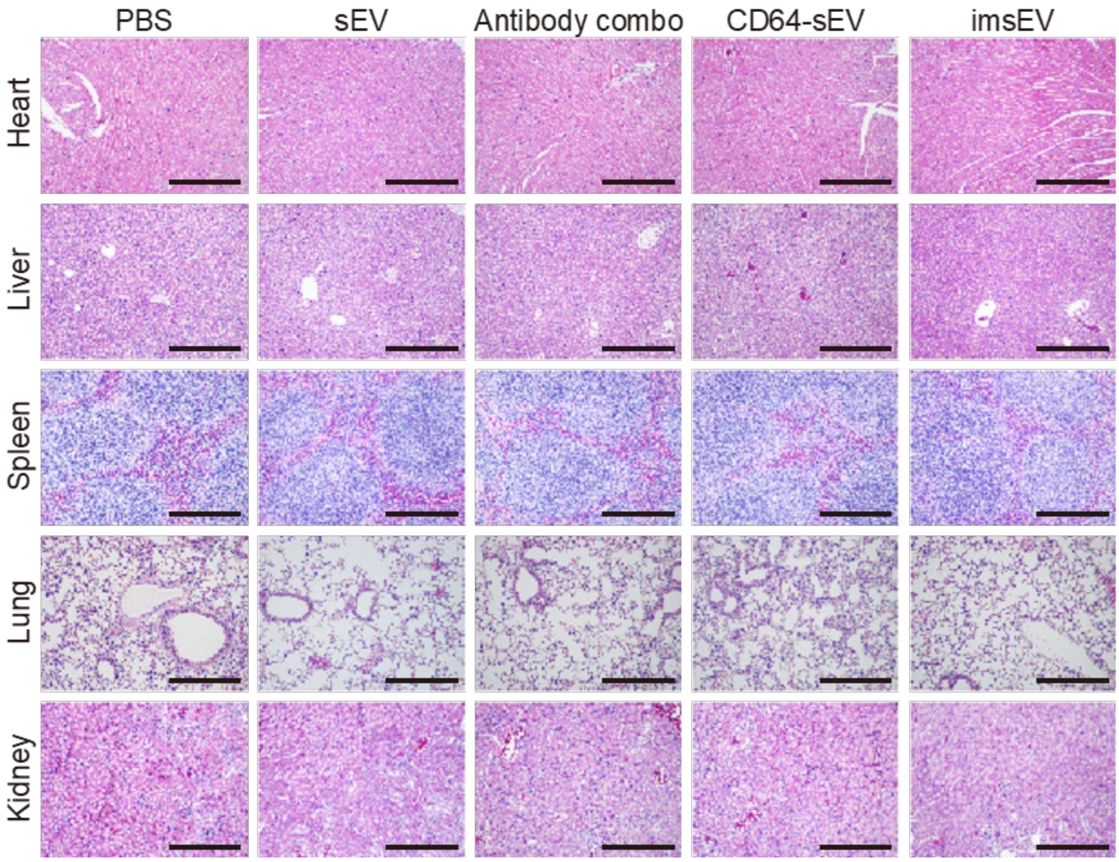

Supplementary Fig. 37 | Biosafety evaluation of imsEV in SB28 model. H&E staining of heart, liver, spleen, lung and kidney tissue after the indicated treatments show that imsEV had no detectable effect on the tissues examined in SB28 model. Scale bar: 500 μm.

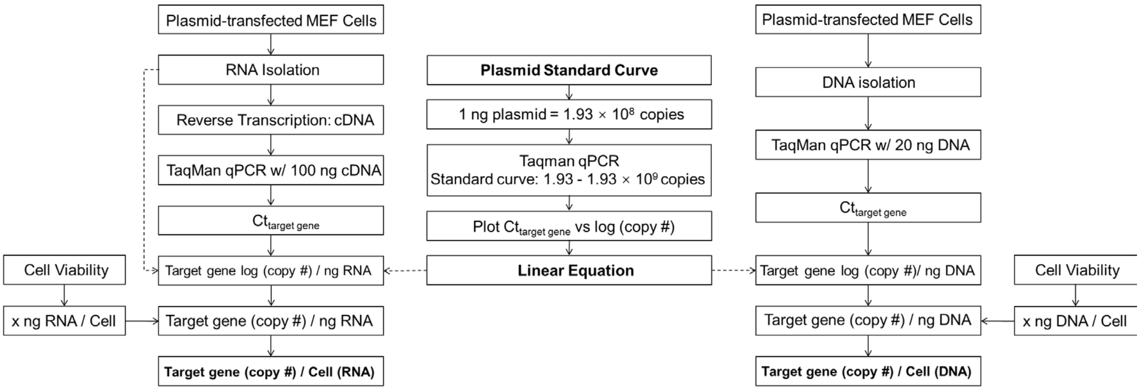

Supplementary Fig. 38 | Flow chart for estimating copy numbers of plasmids and subsequently transcribed mRNA in transfected cells.

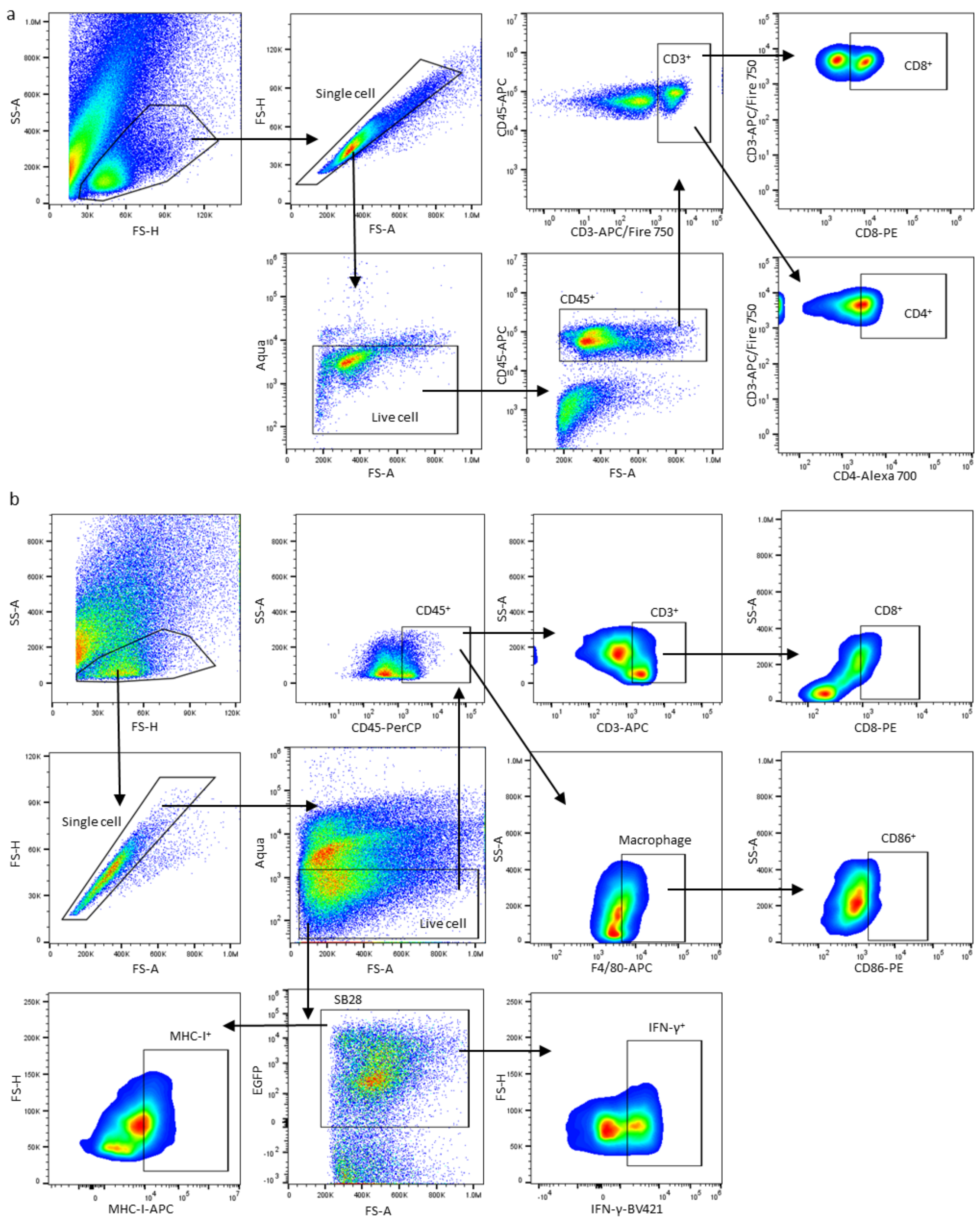

Supplementary Fig. 39 | Representative flow cytometry gating strategies. a, Representative gating for data analyzed in Supplementary Fig. 23, 24, 34, and 35. **b,** Representative gating for data analyzed in Supplementary Fig. 25, 28a, 29a, 30a, and 31a.

SUPPLEMENTARY TABLES

Supplementary Table 1 | Controlling sEV release from MEFs by adjusting the amplitude of nanosecond pulses. Values shown are means ± SD (n = 3 biologically independent samples).

Amplitude, V	Duration, ns	Frequency, Hz	Cell viability, %	sEV numbers per cell, $\times 10^4$
0	600	100k	78.87 ± 1.41	6.10 ± 0.55
50	600	100k	78.28 ± 2.50	6.67 ± 0.65
100	600	100k	79.38 ± 2.34	47.38 ± 5.42
150	600	100k	78.47 ± 4.55	61.78 ± 5.24
180	600	100k	77.06 ± 3.76	63.66 ± 2.48
200	600	100k	65.69 ± 2.37	63.18 ± 3.99
250	600	100k	58.04 ± 5.13	62.41 ± 6.65

Supplementary Table 2 | Controlling sEV release from MEFs by adjusting the duration of nanosecond pulses. Values shown are means ± SD (n = 3 biologically independent samples).

Amplitude, V	Duration, ns	Frequency, Hz	Cell viability, %	sEV numbers per cell, $\times 10^4$
180	300	100k	51.73 ± 4.94	41.61 ± 3.40
180	600	100k	78.04 ± 8.76	62.71 ± 4.21
180	900	100k	71.51 ± 2.52	52.11 ± 10.65
180	1200	100k	48.79 ± 8.47	38.81 ± 2.91
180	1500	100k	28.69 ± 10.19	20.95 ± 4.59

Supplementary Table 3 | Controlling sEV release from MEFs by adjusting the frequency of nanosecond pulses. Values shown are means ± SD (n = 3 biologically independent samples).

Amplitude, V	Duration, ns	Frequency, Hz	Cell viability, %	sEV numbers per cell, ×10^4
180	600	10k	9.84 ± 3.22	23.22 ± 6.45
180	600	100k	76.33 ± 8.13	64.53 ± 7.07
180	600	200k	28.85 ± 7.09	43.59 ± 8.48
180	600	400k	4.07 ± 2.46	2.02 ± 1.35

Supplementary Table 4 | Controlling sEV release from HEK293T cells by adjusting the amplitude of nanosecond pulses. Values shown are means ± SD (n = 3 biologically independent samples).

Amplitude, V	Duration, ns	Frequency, Hz	Cell viability, %	sEV numbers per cell, ×10^4
0	600	100k	80.91 ± 4.37	7.90 ± 0.89
50	600	100k	82.53 ± 5.61	8.08 ± 1.14
100	600	100k	78.41 ± 5.02	58.12 ± 16.24
150	600	100k	79.63 ± 6.36	83.62 ± 6.61
200	600	100k	72.68 ± 4.53	84.03 ± 14.53
250	600	100k	70.01 ± 3.06	81.62 ± 10.26

Supplementary Table 5 | Controlling sEV release from HEK293T cells by adjusting the duration of nanosecond pulses. Values shown are means ± SD (n = 3 biologically independent samples).

**Amplitude, V**	**Duration, ns**	**Frequency, Hz**	**Cell viability, %**	**sEV numbers per cell, ×10^4**
150	300	100k	40.10 ± 3.46	51.96 ± 10.00
150	600	100k	81.96 ± 8.85	85.34 ± 6.24
150	900	100k	60.69 ± 8.79	80.32 ± 6.85
150	1200	100k	51.11 ± 6.37	72.42 ± 7.16
150	1500	100k	15.37 ± 4.81	25.78 ± 8.60

Supplementary Table 6 | Controlling sEV release from HEK293T cells by adjusting the frequency of nanosecond pulses. Values shown are means ± SD (n = 3 biologically independent samples).

**Amplitude, V**	**Duration, ns**	**Frequency, Hz**	**Cell viability, %**	**sEV numbers per cell, ×10^4**
150	600	10k	13.10 ± 4.29	30.10 ± 8.52
150	600	100k	79.35 ± 8.93	82.29 ± 6.24
150	600	200k	24.78 ± 10.43	36.38 ± 6.04
150	600	400k	2.63 ± 1.69	2.19 ± 1.51

Supplementary Table 7 | Changes in sEV-related proteins in MEFs after nsEP treatment. Statistical significance was calculated using a two-sided Student's *t*-test when the data met the assumptions of equal variance or Welch's *t*-test for cases with heterogeneity of variance.

Gene	Fold change	*P* value	Gene	Fold change	*P* value
Serpinb6	46.791	6.75E-03	Dag1	1.685	3.83E-04
Psap	17.377	9.09E-04	Alb	1.683	9.36E-05
Plg	14.484	2.72E-02	Sord	1.607	1.28E-03
Park7	13.116	8.08E-04	B2m	1.606	4.72E-05
Sod1	7.567	9.21E-04	Ddt	1.606	2.84E-04
Rpl39	6.775	3.69E-03	Acy1	1.581	5.48E-03
Tsku	5.279	4.02E-03	Gla	1.575	1.40E-02
Ptx3	5.125	8.51E-03	Anpep	1.532	5.37E-06
Ptgis	4.822	1.74E-03	Dicer1	1.523	7.91E-03
Pgk1	4.727	5.24E-04	Ago2	1.514	4.61E-02
Rbmxl1	4.724	3.29E-02	Aga	1.511	1.31E-02
Serpinb1a	4.244	1.56E-04	Fam20a	0.664	2.45E-02
Ide	4.126	5.99E-04	Htra1	0.662	4.44E-04
Pebp1	4.093	6.90E-05	Gnl1	0.654	1.25E-04
Serpinf1	3.773	1.52E-02	Ecm1	0.639	2.67E-03
Ppt1	3.512	4.94E-03	Aldoa	0.623	1.65E-03
Tfrc	3.499	8.48E-05	Akr1b1	0.602	1.82E-03
Axl	3.488	7.88E-05	Apob	0.599	9.65E-04
Vasn	3.473	1.28E-02	Asah1	0.591	7.02E-04
Mmp3	3.402	6.60E-04	Grem1	0.588	1.92E-02
H2-K1	3.213	9.51E-07	Lgals8	0.584	3.17E-03
Thbs1	3.095	7.19E-04	Lgals3	0.578	1.03E-03
Vcam1	3.021	1.29E-03	Man2b1	0.568	3.99E-03
Yars1	2.99	3.99E-03	Ogn	0.568	2.47E-02
Sdcbp	2.796	4.75E-03	Il1rn	0.568	2.28E-09
Sri	2.734	3.21E-02	Loxl3	0.524	4.45E-03
Qsox1	2.589	5.32E-03	Il6st	0.492	1.23E-04
Saal1	2.459	4.02E-03	Gsn	0.482	3.61E-04
Aebp1	2.366	7.53E-06	Lgals3bp	0.46	5.31E-04
Cstb	2.335	3.63E-03	Itm2c	0.442	4.21E-05

Ada	2.312	7.95E-03	Lgals1	0.439	1.45E-03
Fabp5	2.309	7.90E-04	Igf2r	0.427	4.12E-04
F5	2.304	3.10E-03	Gars1	0.42	1.17E-03
H2-D1	2.283	2.59E-06	H2bc3	0.417	7.33E-03
Adam9	2.248	3.60E-04	Lox	0.408	2.33E-04
Clic1	2.222	4.82E-04	Grn	0.397	2.90E-03
Col1a1	2.211	6.90E-04	Pcmt1	0.396	2.81E-02
Adam15	2.146	8.64E-03	Mif	0.373	2.77E-03
App	2.131	4.62E-05	Alad	0.36	6.44E-04
Gusb	2.124	8.13E-04	Tgfb2	0.348	1.17E-03
Apoa1	2.086	7.00E-04	Tpt1	0.344	1.77E-03
Anxa1	2.038	6.24E-04	Xdh	0.338	3.28E-05
Clec2d	2.029	6.30E-04	Wls	0.307	2.37E-03
Cat	2.017	1.27E-02	Cx3cl1	0.274	1.50E-03
Anxa5	2.014	1.02E-04	Mmp14	0.267	3.16E-03
Cpq	2.002	1.05E-03	Lcn2	0.252	1.30E-04
Ctsb	1.985	6.91E-04	Gsdmd	0.237	6.37E-04
Rnpep	1.956	2.88E-03	Ltf	0.183	5.48E-04
Ctsd	1.852	5.11E-04	Cpe	0.144	1.98E-03
Glb1	1.839	6.40E-04	Serpine2	0.123	2.43E-04
Fam20c	1.821	4.79E-03	Arhgdia	0.116	1.65E-03
C4b	1.783	4.00E-05	Fn1	0.073	2.58E-04

Supplementary Table 8 | Differentially expressed proteins associated with sEV secretion based on protein-protein interaction analysis. Statistical significance was calculated using a two-sided Student's *t*-test when the data met the assumptions of equal variance or Welch's *t*-test for cases with heterogeneity of variance.

Gene	Fold change	*P* value	Gene	Fold change	*P* value
MLKL	81.37	8.67E-07	Rab8a	0.613	0.007538
Sdcbp	0.396	0.004749	Rab27b	0.229	0.001798
Sdc2	0.167	0.001291	Rala	0.573	0.003197
Ndfip1	0.199	0.000999	Isg15	189.029	8.13E-07
Vps36	1.822	0.001329	Usp18	25.942	0.000123
Vta1	1.659	0.001819	Prnp	0.169	0.001339
Chmp4b	3.245	0.014477	Stx17	0.396	0.026902
Mvb12a	0.596	0.041535	Vamp7	0.593	0.005262
Chmp3	1.807	0.029396	Ykt6	2.773	0.000851
Chmp5	1.712	0.035884	Vamp8	0.656	0.012828
Vta1	1.659	0.001819			

Supplementary Table 9 | Top 95 proteins differentially expressed after nsEP treatment.

Statistical significance was calculated using a two-sided Student's *t*-test when the data met the assumptions of equal variance or Welch's *t*-test for cases with heterogeneity of variance.

Gene	Fold change	*P* value	Gene	Fold change	*P* value
Ifit3	443.133	8.28E-07	Ddx58	9.185	1.72E-06
Phf11	221.3	9.29E-05	Mrps25	8.763	2.63E-04
Isg15	189.029	8.13E-07	Tapbp	8.757	4.26E-06
Cmpk2	132.26	8.44E-04	Tubb1	8.361	2.01E-04
Ifih1	122.537	1.18E-06	Tap1	8.186	1.87E-05
Iigp1	120.255	1.57E-06	Nnmt	8.075	5.85E-03
Gbp2	117.786	1.84E-06	NA	7.888	3.30E-04
Ifit2	104.698	5.91E-05	Slfn9	7.701	1.51E-05
Gbp4	89	4.24E-05	Tgm2	7.68	6.41E-09
MLKL	81.37	8.67E-07	Ifi204	7.613	8.73E-06
Tgtp2	53.508	6.51E-06	Ltf	7.567	5.48E-04
Oas1a	49.733	1.58E-05	Ldhb	7.566	2.33E-05
H2-T23	48.97	1.79E-04	Cth	7.433	4.99E-05
Lgals3bp	46.791	5.31E-04	Adar	7.219	6.19E-04
Dtx3l	39.042	1.28E-03	Irgm1	6.961	8.96E-06
Oasl1	32.668	1.22E-05	Anpep	6.775	5.37E-06
Usp18	25.942	1.23E-04	Fabp4	6.632	2.09E-05
Ric8a	24.922	5.41E-06	Steap4	6.575	5.02E-03
Dhx58	23.254	3.54E-04	Ifitm3	6.532	4.04E-05
Samd9l	22.422	2.24E-05	Rpe	6.509	6.89E-04
Parp14	22.105	4.99E-04	Epdr1	6.443	5.15E-04
Mnda	21.861	4.30E-06	Msi2	6.168	5.26E-04
Psmb10	20.145	1.45E-05	Sdc2	0.167	1.29E-03
Psmb9	19.11	2.36E-05	Ssbp2	0.161	4.85E-05
Bst2	18.538	1.05E-06	Lrrc32	0.158	5.99E-05
Aldh1a7	18.041	4.48E-05	Trps1	0.15	3.99E-03
Trim30a	17.903	2.26E-06	Mmp3	0.144	6.60E-04
Ifit1	17.402	2.99E-07	Ndfip2	0.14	4.94E-02
Il1rn	17.377	2.28E-09	Setd1a	0.139	1.16E-02
Stat2	17.33	1.43E-05	Gja1	0.139	1.24E-05
Stat1	16.114	1.23E-07	Zc3h18	0.136	2.13E-02
Arhgdib	15.53	4.77E-04	Nrp2	0.132	2.82E-03
Isg20	15.295	8.98E-08	Vasn	0.123	1.28E-02
Plin2	14.859	9.11E-05	Zdhhc20	0.121	4.06E-03

Parp10	14.506	7.34E-06	Med14	0.121	3.54E-03
Alb	14.484	9.36E-05	Cx3cl1	0.116	1.50E-03
Trex1	13.65	4.70E-05	Col5a1	0.105	6.59E-05
Sts	13.446	6.99E-06	Ubtf	0.092	1.94E-02
Zbp1	13.367	3.31E-03	Trpv4	0.088	1.75E-03
H2-K1	13.116	9.51E-07	Fbln2	0.088	3.00E-06
Daxx	13.033	3.59E-02	Efnb2	0.087	1.94E-04
Gstm2	12.362	3.48E-04	Loxl4	0.074	3.48E-04
Ifi35	11.674	2.73E-05	Apob	0.073	9.65E-04
Parp9	11.539	1.77E-07	Topbp1	0.07	1.73E-02
Tor3a	11.476	1.12E-03	Ccn1	0.054	6.72E-05
Ifi44	11.205	1.34E-05	Cspg4	0.048	1.66E-03
Pyhin1	9.965	1.40E-05	Ldlr	0.015	7.92E-06
Psmb8	9.882	4.28E-06			

Supplementary Table 10 | sEVs incubated with the indicated antibody ratios of monoclonal anti-CD71 and anti-PDL1 antibodies, followed by measurement of binding of imsEV to anti-CD71 and anti-PDL1 antibodies by tethered lipoplex nanoparticle (TLN) technology. Values shown are means ± SD (n = 3 biologically independent samples).

anti-CD71: anti-PD-L1	Colocalization of sEV/anti-CD71	Colocalization of sEV/anti-PD-L1	Colocalization of sEV/anti-CD71/anti-PD-L1
1: 5	0.83 ± 0.01	0.80 ± 0.06	0.69 ± 0.09
1: 3	0.88 ± 0.03	0.79 ± 0.03	0.72 ± 0.11
1: 1	0.92 ± 0.04	0.68 ± 0.07	0.61 ± 0.08
3: 1	0.95 ± 0.04	0.58 ± 0.01	0.55 ± 0.12
5: 1	0.96 ± 0.02	0.43 ± 0.03	0.41 ± 0.06
