# Supplementary material for: Adaptive design of mRNA-loaded extracellular vesicles for targeted immunotherapy of cancer
Source: Nat Commun. 2023 Oct 19;14:6610. doi: 10.1038/s41467-023-42365-5 (PMC10587228; doi:10.1038/s41467-023-42365-5)
Supplement: Supplementary file 3 — Reporting Summary [file 41467_2023_42365_MOESM3_ESM.pdf]

nature portfolio

Corresponding author(s): Shengnian Wang, Lesheng Teng, Betty Y.S. Kim, Zhaogang Yang, Wen Jiang

Last updated by author(s): Sep 9, 2023

Reporting Summary

Nature Portfolio wishes to improve the reproducibility of the work that we publish. This form provides structure for consistency and transparency in reporting. For further information on Nature Portfolio policies, see our Editorial Policies and the Editorial Policy Checklist.

Statistics

For all statistical analyses, confirm that the following items are present in the figure legend, table legend, main text, or Methods section.

n/a	Confirmed	
☐	☒	The exact sample size (*n*) for each experimental group/condition, given as a discrete number and unit of measurement
☐	☒	A statement on whether measurements were taken from distinct samples or whether the same sample was measured repeatedly
☐	☒	The statistical test(s) used AND whether they are one- or two-sided<br>*Only common tests should be described solely by name; describe more complex techniques in the Methods section.*
☐	☒	A description of all covariates tested
☐	☒	A description of any assumptions or corrections, such as tests of normality and adjustment for multiple comparisons
☐	☒	A full description of the statistical parameters including central tendency (e.g. means) or other basic estimates (e.g. regression coefficient) AND variation (e.g. standard deviation) or associated estimates of uncertainty (e.g. confidence intervals)
☐	☒	For null hypothesis testing, the test statistic (e.g. *F*, *t*, *r*) with confidence intervals, effect sizes, degrees of freedom and *P* value noted<br>*Give P values as exact values whenever suitable.*
☒	☐	For Bayesian analysis, information on the choice of priors and Markov chain Monte Carlo settings
☒	☐	For hierarchical and complex designs, identification of the appropriate level for tests and full reporting of outcomes
☒	☐	Estimates of effect sizes (e.g. Cohen's *d*, Pearson's *r*), indicating how they were calculated

Our web collection on statistics for biologists contains articles on many of the points above.

Software and code

Policy information about availability of computer code

Data collection	Living Image Software 4.7, ZEN 2.3, Accuri C6 Plus Software v1.0.23.1, TIRF microscopy, Cytek@Aurora, Nanoparticle Tracking Analysis (NTA) software 3.40
Data analysis	GraphPad Prism 9.0.0, FlowJo 10.7.2, Microsoft Excel 2021, Proteome Discoverer v2.4.1.15, Cytoscape v3.7.2, Image J 1.53t, Living Image Software 4.7

For manuscripts utilizing custom algorithms or software that are central to the research but not yet described in published literature, software must be made available to editors and reviewers. We strongly encourage code deposition in a community repository (e.g. GitHub). See the Nature Portfolio guidelines for submitting code & software for further information.

Data

Policy information about availability of data

All manuscripts must include a data availability statement. This statement should provide the following information, where applicable:

- Accession codes, unique identifiers, or web links for publicly available datasets
- A description of any restrictions on data availability
- For clinical datasets or third party data, please ensure that the statement adheres to our policy

The authors declare that data supporting the findings of this study are available within the article and its Supplementary Information and the Source data file. Source data file are provided with this paper. All equipment and reagents are commercially available and are described in the Methods section.

Human research participants

Policy information about studies involving human research participants and Sex and Gender in Research.

Reporting on sex and gender	N/A
Population characteristics	N/A
Recruitment	N/A
Ethics oversight	N/A

Note that full information on the approval of the study protocol must also be provided in the manuscript.

Field-specific reporting

Please select the one below that is the best fit for your research. If you are not sure, read the appropriate sections before making your selection.

[x] Life sciences [ ] Behavioural & social sciences [ ] Ecological, evolutionary & environmental sciences

For a reference copy of the document with all sections, see nature.com/documents/nr-reporting-summary-flat.pdf

Life sciences study design

All studies must disclose on these points even when the disclosure is negative.

Sample size	No effect size was predetermined. The sample sizes were kept consistent with previously published studies where in vitro studies were repeated at least three times independently. For in vivo experiments, at least 3 mice per group were used, which was estimated to achieve the detection of significant differences between groups based on means and standard deviations. Details regarding the sample size of all experiments are provided in figure legends.
Data exclusions	No data were excluded from final analyses.
Replication	Three replicates were performed for each experiment with similar results. Data are shown as mean with standard deviation. All attempts at replication were successful.
Randomization	All samples were randomized before experiments. For in vivo studies, randomization was used to divide up the animals for in vivo treatment study.
Blinding	The investigators and authors have been consistently blinded to the group allocation during data collection and analysis.

Reporting for specific materials, systems and methods

We require information from authors about some types of materials, experimental systems and methods used in many studies. Here, indicate whether each material, system or method listed is relevant to your study. If you are not sure if a list item applies to your research, read the appropriate section before selecting a response.

Materials & experimental systems

n/a	Involved in the study	
[ ]	[x]	Antibodies
[ ]	[x]	Eukaryotic cell lines
[x]	[ ]	Palaeontology and archaeology
[ ]	[x]	Animals and other organisms
[x]	[ ]	Clinical data
[x]	[ ]	Dual use research of concern

Methods

n/a	Involved in the study	
[x]	[ ]	ChIP-seq
[ ]	[x]	Flow cytometry
[ ]	[x]	MRI-based neuroimaging

Antibodies

Antibodies used	Flow cytometry:<br>anti-CD16/CD32 antibody, Bio X Cell, Cat # BE0307, dilution 1:200; Manufacturer verified application is in vitro Fc receptor blocking.<br>https://bioxcell.com/invivomab-anti-mouse-cd16-cd32

anti-CD3-APC/Fire™ 750 antibody, BioLegend, Cat # 100248, dilution 1:200; Manufacturer verified application is flow cytometry (flow). Reactive species is mouse.
https://www.biolegend.com/en-us/products/apc-fire-750-anti-mouse-cd3-antibody-13052

anti-CD3-APC antibody, BioLegend, Cat # 100236, dilution 1:300; Manufacturer verified application is flow cytometry (flow). Reactive species is mouse.
https://www.biolegend.com/en-us/products/apc-anti-mouse-cd3-antibody-8055

anti-CD8a-PE antibody, BioLegend, Cat # 100708, dilution 1:200; Manufacturer verified application is flow. Reactive species is mouse.
https://www.biolegend.com/en-us/products/pe-anti-mouse-cd8a-antibody-155

anti-CD4-Alexa Fluor® 700 antibody, BioLegend, Cat # 100430, dilution 1:200; Manufacturer verified application is flow. Reactive species is mouse.
https://www.biolegend.com/en-us/products/alexa-fluor-700-anti-mouse-cd4-antibody-3385

anti-CD86-PE antibody, BioLegend, Cat # 105008, dilution 1:300; Manufacturer verified application is flow. Reactive species is mouse.
https://www.biolegend.com/en-us/products/pe-anti-mouse-cd86-antibody-256

anti-IFN-γ-BV421 antibody, BioLegend, Cat # 505829, dilution 1:200; Manufacturer verified application is flow. Reactive species is mouse.
https://www.biolegend.com/en-us/products/brilliant-violet-421-anti-mouse-ifn-gamma-antibody-7154

anti-H-2Kd/H-2Dd-APC antibody, BioLegend, Cat # 114714, dilution 1:200; Manufacturer verified application is flow. Reactive species is mouse.
https://www.biolegend.com/en-us/products/apc-anti-mouse-h-2kd-h-2dd-antibody-15819

anti-CD45-APC antibody, BioLegend, Cat # 147708, dilution 1:200; Manufacturer verified application is flow. Reactive species is mouse.
https://www.biolegend.com/en-us/products/apc-anti-mouse-cd45-antibody-9795

anti-CD45-PerCP antibody, BioLegend, Cat # 103130, dilution 1:200; Manufacturer verified application is flow. Reactive species is mouse.
https://www.biolegend.com/en-us/products/percp-anti-mouse-cd45-antibody-4265

anti-F4/80-APC antibody, BioLegend, Cat # 123116, dilution 1:300; Manufacturer verified application is flow. Reactive species is mouse.
https://www.biolegend.com/en-us/products/apc-anti-mouse-f4-80-antibody-4071

Western blotting and Immunostaining:
anti-MLKL antibody, Abcam, Cat # ab243142, dilution 1:1000; Manufacturer verified application is WB. Reactive species are mouse, rat, and human.
https://www.abcam.com/products/primary-antibodies/mlkl-antibody-3h1-ab243142.html

anti-β-Actin antibody, Cell Signaling Technology, Cat # 4967, dilution 1:3000; Manufacturer verified application is WB. Reactive species are mouse, rat, and human.
https://www.cellsignal.com/products/primary-antibodies/b-actin-antibody/4967

anti-CD64 antibody, ThermoFisher Scientific, Cat # MA5-29706, dilution 1:1000; Manufacturer verified application is WB. Reactive species is mouse.
https://www.thermofisher.com/antibody/product/CD64-Antibody-clone-27-Recombinant-Monoclonal/MA5-29706

anti-CD81 antibody, ThermoFisher Scientific, Cat # MA5-32333, dilution 1:1000; Manufacturer verified application is WB. Reactive species are mouse, rat, and human.
https://www.thermofisher.com/antibody/product/CD81-Antibody-clone-SN206-01-Recombinant-Monoclonal/MA5-32333

anti-CD9 antibody, ThermoFisher Scientific, Cat # MA5-31980, dilution 1:1000; Manufacturer verified application is WB. Reactive species are mouse, rat, and human.
https://www.thermofisher.com/antibody/product/CD9-Antibody-clone-SA35-08-Recombinant-Monoclonal/MA5-31980

anti-CD63 antibody, ThermoFisher Scientific, Cat # MA5-35208, dilution 1:1000; Manufacturer verified application is WB. Reactive species are mouse, rat, and human.
https://www.thermofisher.com/antibody/product/CD63-Antibody-clone-2H5I1-Recombinant-Monoclonal/MA5-35208

anti-CD71 antibody, Abcam, Cat # ab84036, dilution 1:1000; Manufacturer verified application is WB. Reactive species are mouse and human.
abcam.com/products/primary-antibodies/transferrin-receptor-antibody-ab84036.html

anti-GAPDH antibody, Cell Signaling Technology, Cat # 2118, dilution 1:2000; Manufacturer verified application is WB. Reactive species are mouse, rat, and human.
https://www.cellsignal.com/products/primary-antibodies/gapdh-14c10-rabbit-mab/2118

anti-MHC-I antibody, Abcam, Cat # ab281902, dilution 1:1000; Manufacturer verified application is WB. Reactive species is mouse.
https://www.abcam.com/products/primary-antibodies/mhc-class-i-antibody-r1-212-rabbit-igg-chimeric-ab281902.html

anti-Thrombospondin 1 antibody, Abcam, Cat # ab267388, dilution 1:1000; Manufacturer verified application is WB. Reactive species are mouse, rat, and human.
https://www.abcam.com/products/primary-antibodies/thrombospondin-1-antibody-epr22927-54-ab267388.html

Immunohistochemistry Staining:
anti-Ki67 antibody, Abcam, Cat # ab15580, dilution 1:500. Manufacturer verified application are IHC-P and ICC/IF. Reactive species are mouse and human.
https://www.abcam.com/products/primary-antibodies/ki67-antibody-ab15580.html

anti-IBA1 antibody, ThermoFisher Scientific, Cat # PA5-27436, dilution 1:100; Manufacturer verified application is ICC/IF. Reactive species are mouse, rat, and human.
https://www.thermofisher.com/antibody/product/IBA1-Antibody-Polyclonal/PA5-27436

anti-CD86 antibody, ThermoFisher Scientific, Cat # 14-0862-82, dilution 1:100; Manufacturer verified application is ICC/IF. Reactive species is mouse.
https://www.thermofisher.com/antibody/product/CD86-B7-2-Antibody-clone-GL1-Monoclonal/14-0862-82

anti-CD8a antibody, ThermoFisher Scientific, Cat # 14-0081-82, dilution 1:100; Manufacturer verified application are WB, Flow, IP and ICC/IF. Reactive species is mouse.
https://www.thermofisher.com/antibody/product/CD8a-Antibody-clone-53-6-7-Monoclonal/14-0081-82

anti-IFN gamma antibody, ThermoFisher Scientific, Cat # MM700, dilution 1:100; Manufacturer verified application are WB, Flow, ELISA, IHC, and ICC/IF. Reactive species are mouse and human.
https://www.thermofisher.com/antibody/product/IFN-gamma-Antibody-clone-XMG1-2-Monoclonal/MM700

Validation	All antibodies are commercially available and have been validated by the manufacturer.

Eukaryotic cell lines

Policy information about cell lines and Sex and Gender in Research

Cell line source(s)	MEF, HEK293T, U138, LN229, and T98G were purchased from ATCC. SB28 and GL261 were purchased from DSMZ. CT-2A, U87, and U251 were purchased from Millipore Sigma Aldrich.
Authentication	The MEF, HEK293T, U138, LN229, and T98G cell lines were morphologically confirmed by ATCC. The CT-2A, U87 and U251 cell lines were verified by Millipore Sigma using STR-PCR. The SB28 and GL261 cell lines were verified by DSMZ using mitochondrial Cytochrome C Oxidase Subunit 1 (COI) DNA barcoding.
Mycoplasma contamination	All cell lines were tested negative for mycoplasma contamination.
Commonly misidentified lines (See ICLAC register)	No misidentified cell lines used in the study.

Animals and other research organisms

Policy information about studies involving animals; ARRIVE guidelines recommended for reporting animal research, and Sex and Gender in Research

Laboratory animals	C57BL/6J mice (Six- to eight-week-old, female, 20 g) were purchased from Jackson Laboratory or Weitong Lihua Experimental Animal Technology Co. and maintained at the animal facility of The University of Texas MD Anderson Cancer Center or Jilin University in isolator cages in a pathogen-free facility in a standard environmentally controlled room with 50% humidity and 22°C temperature under a 12-12 h light-dark cycle. Standard water and diet were offered for the mice.
Wild animals	N/A
Reporting on sex	Female
Field-collected samples	N/A
Ethics oversight	This research complies with all relevant ethical regulations. All experimental procedures were performed in compliance with the institutional policies and approved protocols of Jilin University (no. SY202110005) or MD Anderson Cancer Center (no. 00002163).

Note that full information on the approval of the study protocol must also be provided in the manuscript.

Flow Cytometry

Plots

Confirm that:

- [x] The axis labels state the marker and fluorochrome used (e.g. CD4-FITC).
- [x] The axis scales are clearly visible. Include numbers along axes only for bottom left plot of group (a 'group' is an analysis of identical markers).
- [x] All plots are contour plots with outliers or pseudocolor plots.
- [x] A numerical value for number of cells or percentage (with statistics) is provided.

Methodology

Sample preparation	To investigate the sEV uptake by tumour cells, PKH26-labeled sEVs were incubated with tumour cells for 4 h. Afterward, the cells were washed three times with cold PBS and then fixed in 4% paraformaldehyde.<br>To assess cell surface and internal antigens, tumour tissues isolated after transcardial perfusion from each treatment group were collected and digested at 37°C for 60 min in 10 mmol/L HEPES buffer with 300 U/mL collagenase D, dispase, and 15 U/mL DNase I to obtain cell suspensions. After dissociation, the cells were filtered through a 70 µm nylon cell strainer and collected. The cells were stained with appropriate antibodies, as described in Methods. Unstained cells and isotype controls were used.
Instrument	Gallios 561, Beckman
Software	Data was analyzed by FlowJo 10.7.2
Cell population abundance	No sorting was performed.
Gating strategy	All gating strategies are provided in the supplementary figures. Cells were initially on a dot plot, SSC-A vs. FSC-A. The negative population was determined by using unstained cells and isotype controls. The population with fluorescence intensity higher than that of the unstained control is considered positive and the other population is considered negative.

- [x] Tick this box to confirm that a figure exemplifying the gating strategy is provided in the Supplementary Information.

Magnetic resonance imaging

Experimental design

Design type	Using small animal MRI to detect brain tumour growth in mice receiving different treatments
Design specifications	*Specify the number of blocks, trials or experimental units per session and/or subject, and specify the length of each trial or block (if trials are blocked) and interval between trials.*
Behavioral performance measures	*State number and/or type of variables recorded (e.g. correct button press, response time) and what statistics were used to establish that the subjects were performing the task as expected (e.g. mean, range, and/or standard deviation across subjects).*

Acquisition

Imaging type(s)	T2-weighted coronal and axial scans
Field strength	Mice were imaged at the MD Anderson Small Animal Imaging Facility using a 7 Tesla (T) 30-cm horizontal bore magnet (Bruker Biospin MRI, Billerica MA).
Sequence & imaging parameters	T2-weighted coronal and axial images [T2-weighted coronal slices with a thickness of 0.75 mm and taken in a field of view (FOV) of 30 x 40, with a matrix size of 256 x 192 pixels, for a resulting in-plane resolution of 0.156 µm; T2-weighted axial slices with a thickness of 0.75 mm and taken in a field of view (FOV) of 30 x 22.5, with a matrix size of 256 x 192 pixels, for a resulting in-plane resolution of 0.117 µm] were acquired using a RARE (rapid acquisition with relaxation enhancement) sequence, with a repetition time (TR) of 3000 ms and an echo time (TE) of 57 ms.
Area of acquisition	Brain

Diffusion MRI [ ] Used [x] Not used

Preprocessing

Preprocessing software	ImageJ
Normalization	*If data were normalized/standardized, describe the approach(es): specify linear or non-linear and define image types used for transformation OR indicate that data were not normalized and explain rationale for lack of normalization.*

Normalization template	*Describe the template used for normalization/transformation, specifying subject space or group standardized space (e.g. original Talairach, MNI305, ICBM152) OR indicate that the data were not normalized.*
Noise and artifact removal	*Describe your procedure(s) for artifact and structured noise removal, specifying motion parameters, tissue signals and physiological signals (heart rate, respiration).*
Volume censoring	*Define your software and/or method and criteria for volume censoring, and state the extent of such censoring.*

Statistical modeling & inference

Model type and settings	*Specify type (mass univariate, multivariate, RSA, predictive, etc.) and describe essential details of the model at the first and second levels (e.g. fixed, random or mixed effects; drift or auto-correlation).*
Effect(s) tested	*Define precise effect in terms of the task or stimulus conditions instead of psychological concepts and indicate whether ANOVA or factorial designs were used.*

Specify type of analysis: ☒ Whole brain ☐ ROI-based ☐ Both

Statistic type for inference (See Eklund et al. 2016)	*Specify voxel-wise or cluster-wise and report all relevant parameters for cluster-wise methods.*
Correction	*Describe the type of correction and how it is obtained for multiple comparisons (e.g. FWE, FDR, permutation or Monte Carlo).*

Models & analysis

n/a	Involved in the study
☒	☐ Functional and/or effective connectivity
☒	☐ Graph analysis
☒	☐ Multivariate modeling or predictive analysis
